# A unique enhancer boundary complex on the mouse ribosomal RNA genes persists after loss of Rrn3 or UBF and the inactivation of RNA polymerase I transcription

**DOI:** 10.1371/journal.pgen.1006899

**Published:** 2017-07-17

**Authors:** Chelsea Herdman, Jean-Clement Mars, Victor Y. Stefanovsky, Michel G. Tremblay, Marianne Sabourin-Felix, Helen Lindsay, Mark D. Robinson, Tom Moss

**Affiliations:** 1 Laboratory of Growth and Development, St-Patrick Research Group in Basic Oncology, Cancer Division of the Quebec University Hospital Research Centre, Québec, Canada; 2 Department of Molecular Biology, Medical Biochemistry and Pathology, Faculty of Medicine, Laval University, Québec, Canada; 3 Institute of Molecular Life Sciences, University of Zürich, Zürich, Switzerland; 4 SIB Swiss Institute of Bioinformatics, University of Zürich, Zürich, Switzerland; National University of Ireland Galway, IRELAND

## Abstract

Transcription of the several hundred of mouse and human Ribosomal RNA (rRNA) genes accounts for the majority of RNA synthesis in the cell nucleus and is the determinant of cytoplasmic ribosome abundance, a key factor in regulating gene expression. The rRNA genes, referred to globally as the rDNA, are clustered as direct repeats at the Nucleolar Organiser Regions, NORs, of several chromosomes, and in many cells the active repeats are transcribed at near saturation levels. The rDNA is also a hotspot of recombination and chromosome breakage, and hence understanding its control has broad importance. Despite the need for a high level of rDNA transcription, typically only a fraction of the rDNA is transcriptionally active, and some NORs are permanently silenced by CpG methylation. Various chromatin-remodelling complexes have been implicated in counteracting silencing to maintain rDNA activity. However, the chromatin structure of the active rDNA fraction is still far from clear. Here we have combined a high-resolution ChIP-Seq protocol with conditional inactivation of key basal factors to better understand what determines active rDNA chromatin. The data resolve questions concerning the interdependence of the basal transcription factors, show that preinitiation complex formation is driven by the architectural factor UBF (UBTF) independently of transcription, and that RPI termination and release corresponds with the site of TTF1 binding. They further reveal the existence of an asymmetric Enhancer Boundary Complex formed by CTCF and Cohesin and flanked upstream by phased nucleosomes and downstream by an arrested RNA Polymerase I complex. We find that the Enhancer Boundary Complex is the only site of active histone modification in the 45kbp rDNA repeat. Strikingly, it not only delimits each functional rRNA gene, but also is stably maintained after gene inactivation and the re-establishment of surrounding repressive chromatin. Our data define a poised state of rDNA chromatin and place the Enhancer Boundary Complex as the likely entry point for chromatin remodelling complexes.

## Introduction

The ability to translate genetic messages into functional proteins is an absolute necessity for life forms on our planet, and is achieved by the largest known enzyme, the ribosome. The mammalian ribosome is a 4 MDa complex of 4 catalytic RNA molecules and more than 80 proteins that uses amino-acid charged transfer RNAs (tRNAs) to decode the genome via the intermediary of the messenger RNAs (mRNAs) [1]. The efficiency of mRNA translation is a key determinant of gene expression. Expression is of course contingent on gene transcription, on RNA processing, transport and degradation, but changes in translational capacity not only control the rate of protein synthesis, but also the spectrum of mRNAs that are translated. In this way translation plays an important role in the regulation of gene expression independently of gene transcription [2–4]. This necessarily also feeds back onto genome programming by determining the available spectrum of transcription and epigenetic factors, as well as on the full spectrum of nuclear and cytoplasmic organelle functions. The importance of differential mRNA translation is evident in the control of cell growth, tumour suppressors, oncogenes and the differentiation of stem cells [4–6]. In short, the ability to translate mRNAs is a key determinant of cellular phenotype, and the central factor in this process is the ribosome. It is therefore essential that we understand the factors that control ribosome synthesis and assembly.

Since a mammalian cell contains up to 10 million ribosomes, e.g. [7, 8], their synthesis in proliferating cells is a major limitation for growth and occupies up to 50% of all gene transcription and a significant proportion of translation [1, 9, 10]. Indeed, ribosome synthesis directly determines cell proliferation, and a doubling of proliferation rate requires a four-fold increase in the ribosome synthesis rate [11]. The major components of the mammalian ribosome, the 18S and 28S ribosomal RNAs (rRNAs) are synthesized along with the 5.8S rRNA as a single 47S rRNA precursor. This precursor rRNA is assembled into pre-ribosomal particles in the nucleolus with the aid of several hundred accessory proteins and hundreds of small guide RNAs, before being processed into the mature rRNAs and transported to the cytoplasm [1, 12, 13].

In human and mouse, the 47S rRNA is encoded on some 200 haploid gene copies that are organized in direct repeats on the short arms of five chromosomes [9, 14, 15]. These large ribosomal DNA (rDNA) loci constitute the Nucleolar Organiser Regions (NORs), and their active transcription is responsible for the assembly of the nucleoli, the largest subnuclear organelle. The 47S rRNA is transcribed by RNA polymerase I (RPI/Polr1/PolI) and a set of basal transcription factors that are dedicated to this task. Three basal RPI factors have been identified, the “Selectivity” complex SL1, containing TBP and TAF1A through D, and the HMG1-box architectural Upstream Binding Factor (UBF) are responsible for forming the pre-initiation complex. The third factor, Rrn3-TIF1A, associates with RPI and is required for recognition of the UBF/SL1 complex at the promoter. Transcription Termination Factor 1 (TTF1), a Reb-homology DNA binding factor related to yeast Reb1 and Nsi1, is required for termination of the 47S rRNA transcript, but also plays a role in determining silencing of the rDNA [16, 17].

Transcription of the rDNA is stimulated in response to nutrients, growth factors and a wide range of cellular stresses, and both Rrn3 and UBF are direct targets of growth regulatory pathways [1, 18–20]. However, a fraction of the rDNA is transcriptionally silent in most somatic cells and cell lines, and some NORs are heterochromatic, probably corresponding to a heavily methylated rDNA subfraction. Various chromatin-remodelling complexes have been implicated in rDNA activity and have been proposed to act by displacing nucleosomes, in particular at the 47S rRNA promoter site. However, the chromatin structure of the active rDNA fraction is still far from clear. Here we have combined a high-resolution ChIP-Seq protocol with conditional inactivation of two key regulated basal factors, Rrn3/TIF1A and UBF to better understand what determines active rDNA chromatin. The data resolve many questions concerning the interdependence and functions of the basal transcription factors and show that UBF defines the chromatin structure of the actively transcribed rDNA. They also answer doubts as to the essential nature of Rrn3/TIF1A *in vivo*. Perhaps most importantly, the data reveal the existence of an Enhancer Boundary Complex formed by CTCF and Cohesin and three or four phased nucleosomes lying immediately adjacent to the Spacer Promoter-Enhancer repeat unit and an arrested RPI elongation complex. We find that this boundary complex is the only significant site of active histone modifications in the whole 45kbp rDNA repeat. Strikingly, the Enhancer Boundary Complex not only clearly delimits the functional rRNA gene unit, but is stably maintained after inactivation of RPI transcription and the replacement of UBF by nucleosomal chromatin. Our data potentially places the Enhancer Boundary Complex as the key entry point for the remodelling complexes that activate rDNA transcription and defines the poised state of rDNA chromatin.

## Results

In order to better understand the factors that determine rRNA gene activity, we first needed to establish high resolution, low noise interaction maps for the components of the RPI transcription machinery. We achieved this using a combination of Chromatin Immuno-Precipitation, massively parallel DNA sequencing (ChIP-Seq) and normalization to sequence coverage obtained on control (input) DNA, see Materials and Methods for details.

### A high-resolution map of basal factors across the mouse rDNA

**Fig 1:**
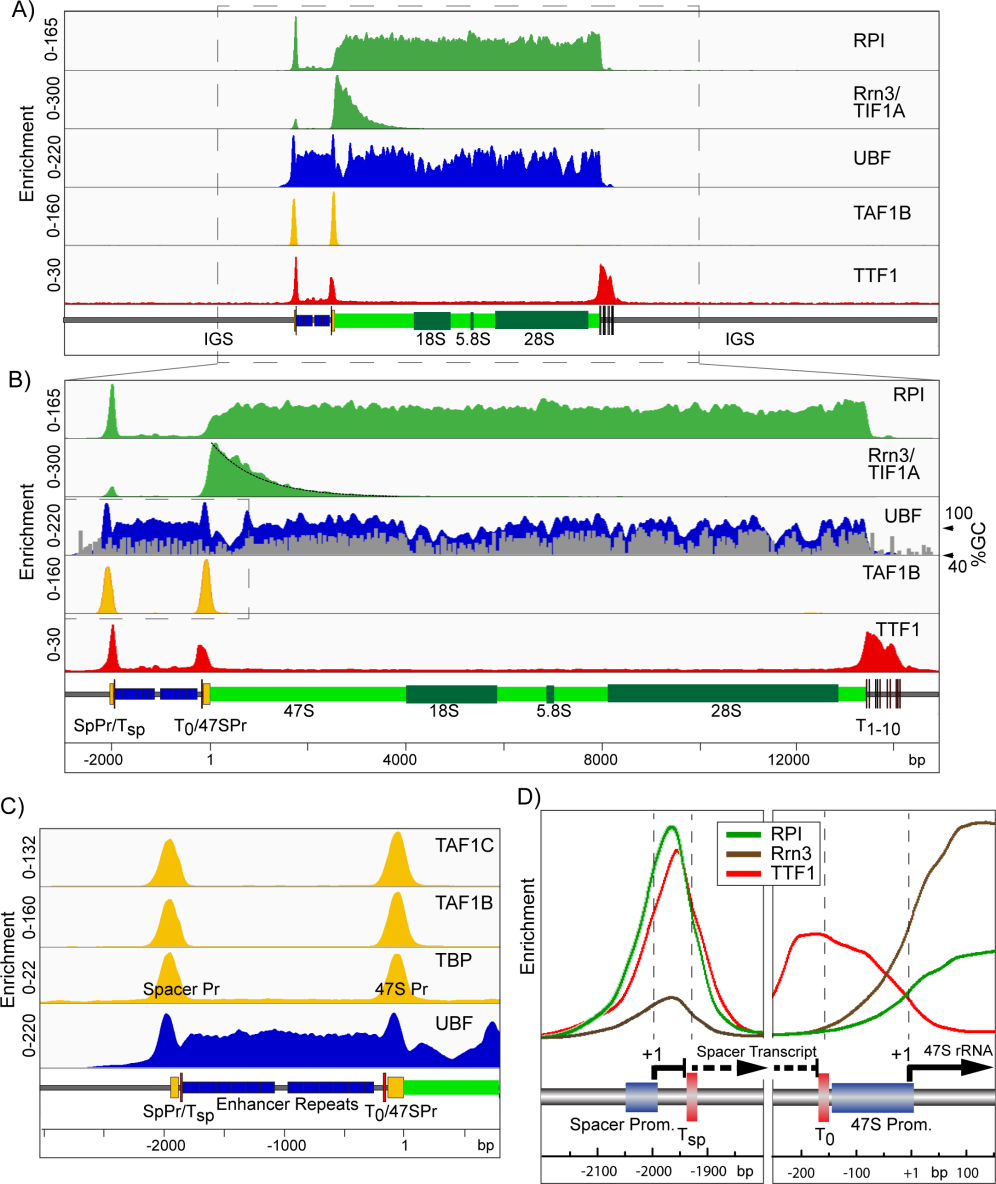
High resolution ChIP-Seq maps of RPI, the RPI basal factors and TTF1 across the mouse rDNA repeat unit of *Ubf*^*+/+*^*/Rrn3*^*+/+*^*p53*^*-/-*^ control MEFs. A) and B) show the ChIP enrichment profile maps for RPI, Rrn3, UBF, the SL1 component TAF1B and TTF1, A) over the full mouse rDNA repeat and, B) at higher resolution for just the functional rRNA gene unit (boxed region in A). The UBF enrichment profile in B) is overlayed with the GC-content profile of the rDNA sequence, see also S1 Fig, and an exponential curve fit to the Rrn3 enrichment profile (black) is shown downstream of the 47S initiation site (+1). C) An enlargement of the enrichment profiles (boxed region in B) for the SL1 components TAF1C, TAF1B and TBP in comparison with that for UBF across the Spacer Promoter, Enhancer Repeat and 47S Promoter. D) Shows the superimposed enrichment profiles of RPI, Rrn3 and TTF1 at the Spacer and 47S Promoters. The enrichment scale for each factor is the same in left and right panels. In A) to D) a scale map of the rDNA sequence elements is given below each panel. ChIP enrichment for each factor is given as; ChIP-Seq reads per million (RPM)/Input DNA RPM.

The normalized ChIP-Seq data from Mouse Embryonic Fibroblasts (MEFs) precisely delineated UBF binding, the SL1 pre-initiation complexes (PICs), engagement of initiation competent and elongating forms of RPI, and the sites of termination factor TTF1 binding (Fig 1). Engagement of RPI was found to be near uniform throughout the 47S transcribed region, strongly suggesting that in MEFs there are no major sites of pausing at which polymerase accumulates. RPI engagement was also found to end abruptly at the downstream TTF1 sites (Fig 1A and 1B), see Discussion. In contrast, the RPI associated initiation factor Rrn3-TIF1A showed an interaction only over the first 2 to 3 kb of the transcribed region, consistent with *in vitro* data showing that Rrn3 is released sometime after initiation [21, 22]. The Rrn3-RPI interaction decayed exponentially with increasing distance from the initiation site, suggesting that its release was stochastic (Fig 1B). Assuming this, and a mean elongation rate of 60 nt.sec^-1^ [23], we could estimate that the half-life of the elongating RPI-Rrn3 complex was around 15 s in MEFs. Thus, Rrn3 acts mechanistically much like the Sigma factors of eubacteria that target the polymerase to promoters but are released during elongation [24–26]. Indeed, like bacterial Sigma, Rrn3 was recently shown to contain a DNA interaction domain that was required for RPI initiation [27]. This said, the recent structural data for RPI-Rrn3 and the RPI-Rrn3-Core Factor complex suggest Rrn3 functions by modulating the RPI structure and dimerization, and that contact between Rrn3 and the RPI promoter DNA is unlikely to occur within the initiation complex [28, 29].

### UBF binding precisely delimits to the functional rDNA unit

Various *in vitro* studies have implicated UBF in the formation of the RPI preinitiation complex [1, 10, 30, 31]. But it has also been implicated in regulating RPI elongation [23] and was shown to bind over a wide region of the rDNA repeat, suggesting a role more in line with the formation of active chromatin [32–35]. Our data now show that in fact UBF is in a position to perform all these roles. UBF was mapped throughout the functional rRNA gene unit and was bounded by two flanking DNA elements, upstream by the Spacer Promoter (SpPr) and downstream by the transcription termination sites (Sal-boxes) bound by Transcription Termination Factor 1 (TTF1). Consistent with its very low sequence selectivity [36–38], UBF bound almost continuously throughout the 47S transcribed region, but displayed a modulation that closely followed the G+C (above 50%) profile of the DNA, (Fig 1B and S1 Fig). This modulation of binding probably represented the true UBF occupancy and was not due to cross-linking or sequencing biases, since it was not observed for RPI. Further, the correlation with high G+C did not hold for the 47S and Spacer Promoter sequences. Both promoters displayed UBF binding that peaked in low G+C sequences and overlapped the SL1/TIF1B TBP-complex binding (TAF1B, -1C and TBP, Fig 1C), defining the promoter PIC. Further, no UBF at all (<1% of 47S region) was detected in the relatively G+C neutral Intergenic Spacer (IGS) (Fig 1A and S1 Fig). Thus, it was very unlikely that recruitment of UBF to the rRNA genes was determined by DNA sequence selectivity. Though once recruited, exact UBF positioning may be affected by underlying DNA sequences and/or the recruitment of other factors such as SL1 and TTF1.

### A stalled RPI transcription complex lies near the upstream UBF boundary

Our previous ChIP data showed a very significant peak of RPI mapping to the Spacer Promoter [35]. The much higher resolution of our ChIP-Seq data revealed that this peak was in fact centred not over the promoter, but 24 bp downstream of the initiation site and just 13 bp upstream of the peak of TTF1 binding associated with the Spacer Termination site T_sp_ (Fig 1D). RPI at this site was also associated with 10 times less Rrn3 than RPI immediately adjacent to the 47S Promoter, and hence was in large part in an initiation incompetent state. Both these observations strongly suggested that transcription complexes initiated at the Spacer Promoter were arrested by TTF1 very early in elongation. The Spacer Promoter was previously shown to be important for the function of the Enhancer repeats [39, 40], which are also thought to be the main entry points for UBF binding [41]. Thus, it was possible that polymerase stalling at the Spacer Promoter played some role in UBF recruitment. Alternatively, it could play a regulatory part in TTF1 induced DNA looping [42] or, by analogy with RNA polymerase II genes, as part of an insulator complex [43].

### Rrn3-TIF1A is an essential factor in mouse

Given the essential nature of UBF in rDNA activity [35], we sought to better understand what defined the extent of its binding and first asked whether rDNA transcription itself was necessary. Rrn3-TIF1A associates with mouse RPI to form the initiation competent form of the polymerase [1, 21, 22] (Fig 1), hence its elimination should specifically inactivate rDNA transcription. However, while the yeast Rrn3 ortholog is essential for rDNA transcription [44], there was some doubt whether this was also the case in mouse [45]. Mouse embryos lacking Rrn3-TIF1A were reported not to arrest development until E9.5, by which time zygotic transcription normally increases rRNA levels by over 1000 fold [46, 47]. Thus, it was possible that mouse Rrn3-TIF1A may not be essential for rDNA transcription. When we analyzed the Rrn3-null mice we found that in fact they arrested development during the early cleavage stages (S2A, S2B and S3A Figs), as previously observed for UBF and RPI deletions. We therefore concluded that mouse Rrn3 was indeed an essential and non-redundant part of the RPI transcription machinery, and hence was an ideal target to induce specific inhibition of rDNA transcription, see legend to S2 Fig for further details.

### RPI transcription is not required to maintain actively poised rDNA

MEFs conditional for Rrn3 (*Rrn3*^*fl/fl*^*/ER-Cre*^*+/+*^*/p53*^*-/-*^) and isogenic control MEFs (*Rrn3*^*+/+*^*/ER-Cre*^*+/+*^*/p53*^*-/-*^) were isolated from embryos carrying the *Rrn3-TIF1A*^*flox*^ allele [45] (S3A Fig, Materials and Methods), and used to determine the rDNA status before and after Rrn3 loss. Treatment of these MEFs with multiple low doses of 4-hydroxytamoxifen (4-HT) inactivated the Rrn3 gene and reduced Rrn3 protein levels by >90%, while the same treatment had no effect on Rrn3 levels in the *Rrn3*^*+/+*^ isogenic control cells (S3B Fig and Materials and Methods). Concomitantly with Rrn3 depletion, *de novo* synthesis of the 47S precursor RNA, as determined by metabolic labelling [48], was strongly suppressed (S3F Fig).

**Fig 2:**
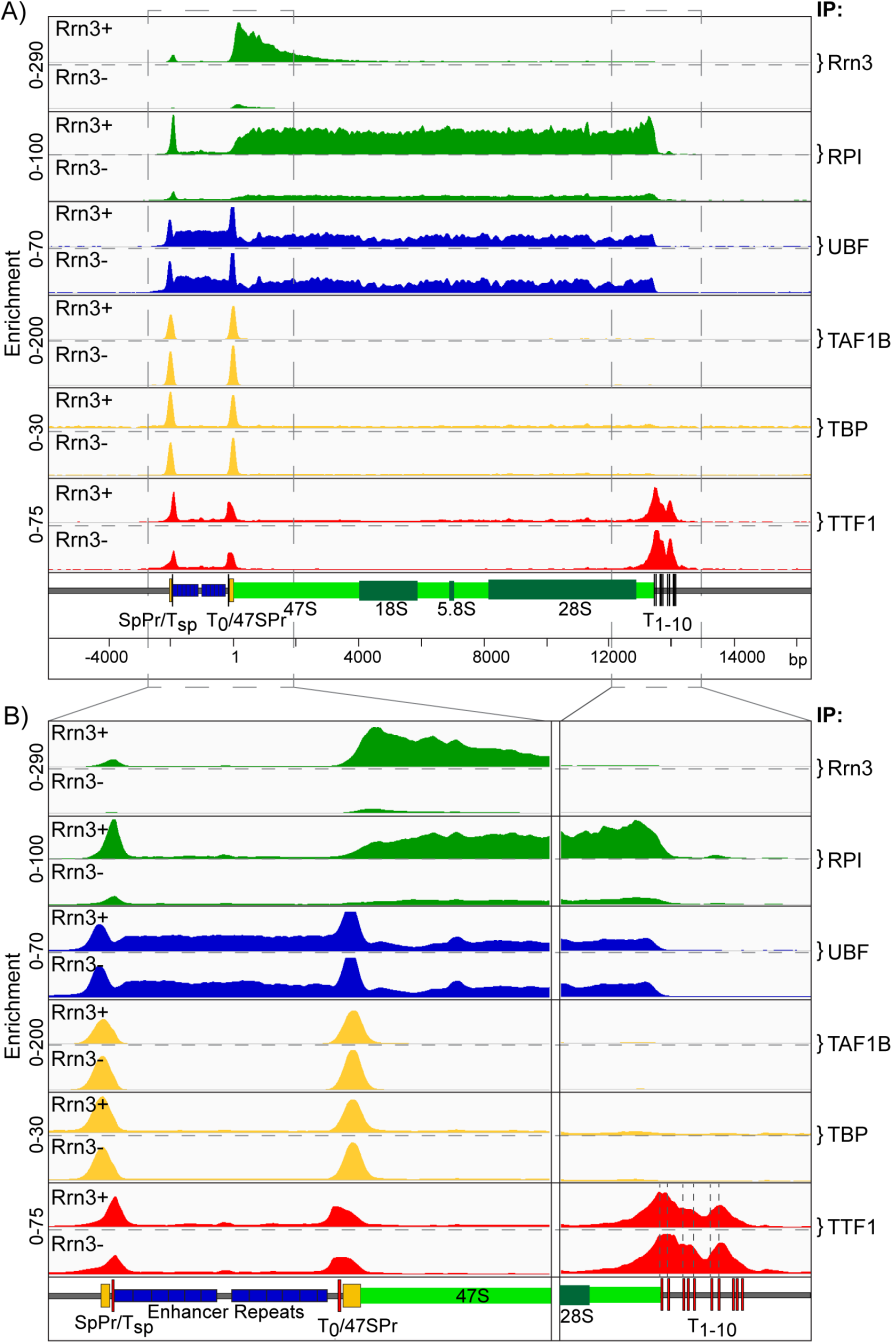
Conditional deletion of the Rrn3 gene inhibits RPI transcription but does not affect pre-initiation complex formation. As in Fig 1., A) and B) show the ChIP enrichment profile maps before (Rrn3+) and after (Rrn3-) Rrn3 gene inactivation (72h 4-HT time point, S3B and S3F Fig). The binding profiles (IP) for Rrn3, RPI, UBF, the SL1 components TAF1B and TBP, and TTF1 are shown in A) over the functional mouse rRNA gene unit and, B) at higher resolution for the upstream Enhancer and Promoter elements and the 47S termination site, (boxed regions in A). As in Fig 1, a scale map of the rDNA sequence elements is given below each panel, and ChIP enrichments for each factor are given as; ChIP-Seq RPM/Input DNA RPM.

High resolution mapping of factor binding across the rDNA repeat in *Rrn3*^*fl/fl*^*/ER-Cre*^*+/+*^*/p53*^*-/-*^ MEFs was indistinguishable from that seen in the wild type MEFs, (compare Figs 1B and 2A). Near complete loss of Rrn3 protein after 4-HT treatment essentially eliminated its engagement with the 5’ of the 47S transcribed region and strongly suppressed recruitment of RPI throughout the 47S region, as well as at the Spacer Promoter (SpPr) (Fig 2A and 2B). In contrast, Rrn3 loss had no effect on the maintenance of UBF binding either within the 47S region, the enhancer repeats, or at the 47S and Spacer Promoters. Binding of the SL1 TBP complex (TAF1B and TBP) at both Promoters was also unaffected by Rrn3 loss, in agreement with data showing Rrn3 is not required to maintain UAF and Core Factor binding at the yeast RPI promoter [49]. Further, TTF1 remained bound at the adjacent T_sp_ and T_0_ sites, as well as at the T_1_-T_10_ 47S termination sites after Rrn3 loss. Parallel 4-HT treatment of isogenic *Rrn3*^*+/+*^*/ER-Cre*^*+/+*^*/p53*^*-/-*^ MEFs had no effect on either Rrn3 or RPI recruitment, or indeed on any of the chromatin factors in this study, see below. Thus, Rrn3 and RPI engagement on the rDNA appeared not to be required for the establishment of the preinitiation complexes at the Spacer and 47S Promoters, or for the normal pattern of UBF binding. In support of this, colony forming assays (S3G Fig) strongly suggested that the remnant engagement of RPI and Rrn3 detected after 4-HT treatment in Fig 2 resulted from a small percentage of cells that had retained a functional Rrn3 gene, and did not represent a low level of transcription in all cells.

These data demonstrated that the potentially active state of the rRNA genes, as defined by SL1-UBF preinitiation complexes at both Spacer and 47S promoters and UBF binding throughout the Enhancer Repeats and 47S gene body, was stably maintained through 48h of very low total Rrn3 levels and 24h of significant transcriptional repression (S3 Fig, panels B and F). This long-term stability of UBF binding was surprising given its low DNA binding constant (Kd ~ 10nM) [50] and high *in vivo* off-rate (t_1/2_ 9 to 25 s) and its inability to compete with nucleosome formation [51]. Thus, the data suggested that a transcription independent mechanism may exist to maintain UBF binding and the potentially active state of the rRNA genes.

### UBF is essential for the recruitment of the RPI transcription machinery

**Fig 3:**
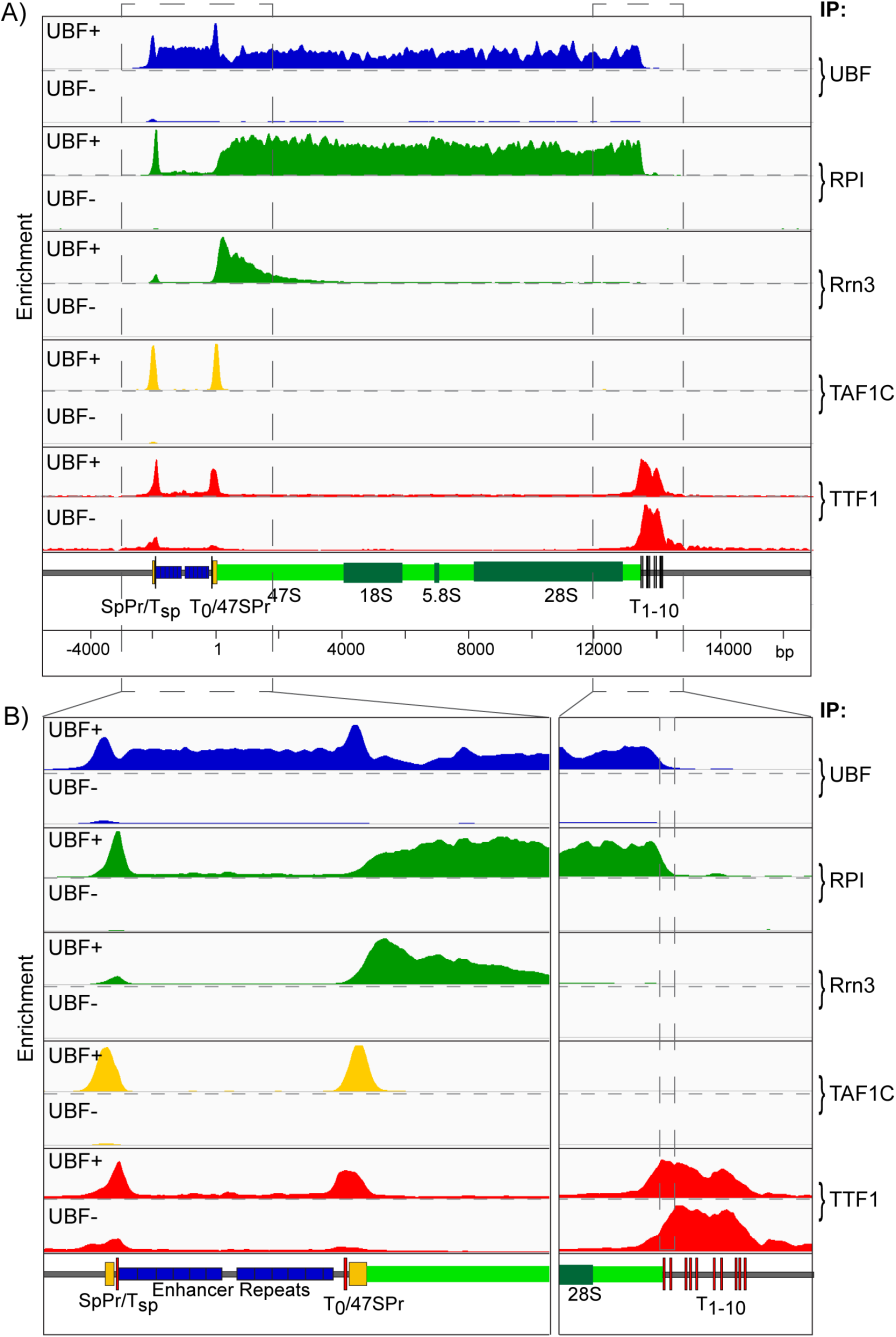
Conditional deletion of the UBF gene not only ablates RPI transcription but also prevents preinitiation complex formation. A) and B) show the ChIP enrichment profile maps before (UBF+) and after (UBF-) UBF gene inactivation (72h 4-HT time point, S3E and S3F Fig). The binding profiles (IP) for UBF RPI, Rrn3, the SL1 component TAF1C, and TTF1 are shown; A) over the functional mouse rRNA gene unit and, B) at higher resolution for the upstream Enhancer and Promoter elements and the 47S termination site. As in Fig 1, a scale map of the rDNA sequence elements is given below each panel, and ChIP enrichments for each factor are given as; ChIP-Seq RPM/Input DNA RPM.

Loss of UBF in SV40 transformed conditional MEFs was previously shown to strongly repress RPI transcription, [35], and this we found also to be the case in the p53^-/-^ immortalized (*Ubf*^*fl/fl*^*/ER-Cre*^*+/+*^*/p53*^*-/-*^) MEFs used in the present study (S3D–S3F Fig). Using these MEFs, and the ChIP-Seq approach, we found that not only was RPI and Rrn3 recruitment eliminated by UBF loss, but also SL1 (e.g. TAF1C) binding at both Spacer and 47S promoters (Fig 3A and 3B). Further, TTF1 binding at the upstream T_sp_ and T_0_ sites was suppressed and there was a small but clear shift in its binding preferences at the 47S termination region, the T_1_ and T_2_ sites being reduced in favour of binding at the downstream sites. This suggested that the domain of UBF binding modulated accessibility of TTF1 to the T_1_ and T_2_ sites. We also consistently observed a significant but low level of TTF recruitment throughout the 47S transcribed region that was significantly reduced on inactivation of RPI transcription whether by loss of Rrn3 or UBF (S4 Fig). This suggested a cycling of TTF1 between the upstream and downstream sites that would be consistent with its rapid dynamics noted previously by Fluorescence Recovery After Photo-bleaching (FRAP) [52]

### UBF determines psoralen accessibility and nucleosome exclusion

**Fig 4:**
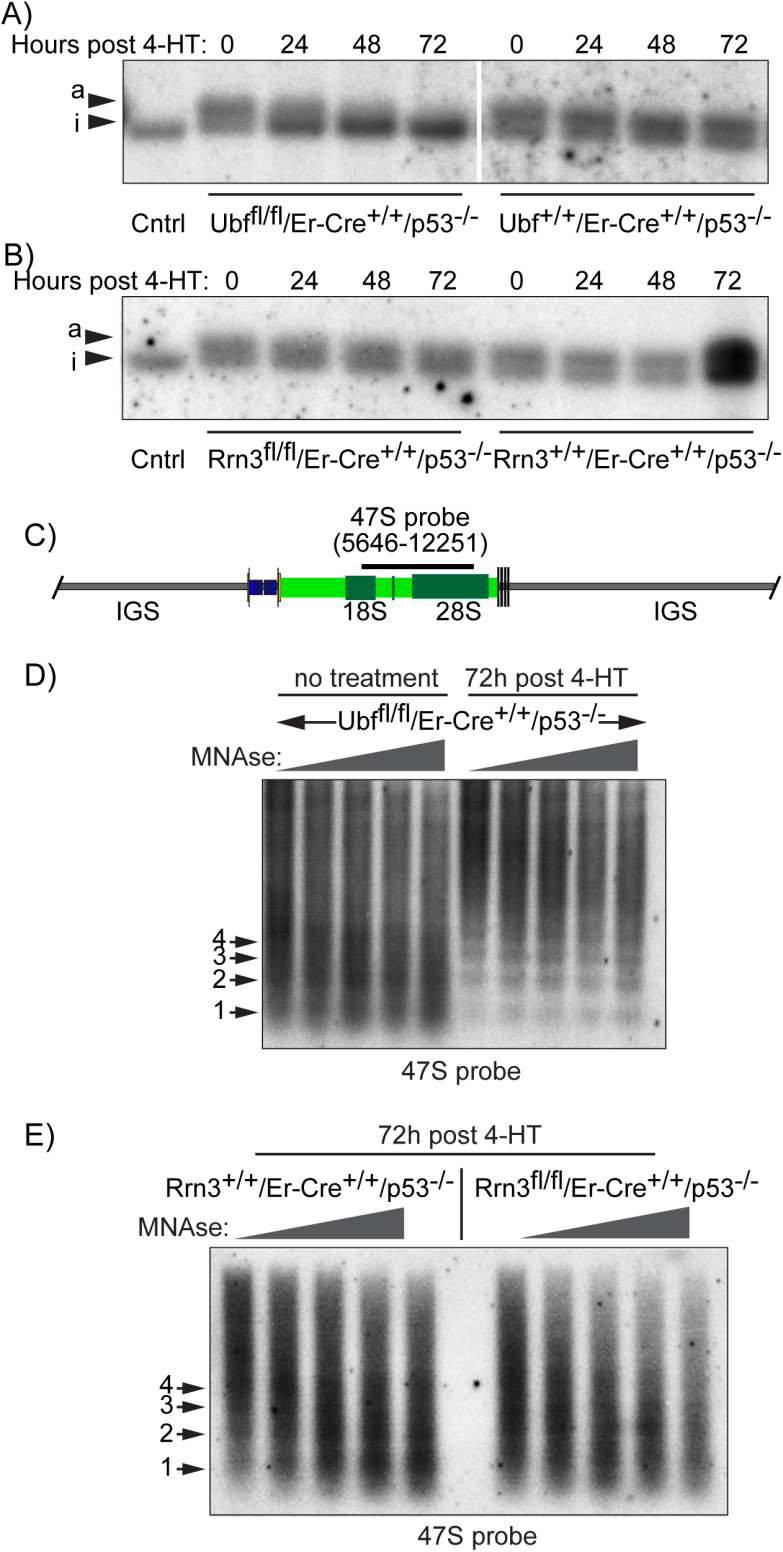
UBF determines the activated state of the rDNA chromatin. A) and B) show psoralen accessibility analyses during the time course of UBF and Rrn3 gene deletion (see 4HT time course S3 Fig). The high accessibility so-called active “a” gene fraction, and the low accessibility inactive. “i” gene fraction were detected using the 47S probe. C) The mapped position of the hybridization probe relative to the start of the 47S rRNA is indicated above a diagram of the rDNA repeat. D) The profiles of increasing MNase cleavage of chromatin from UBF conditional MEFs either before or after (72h post 4-HT) inactivation of the UBF gene. E) The profiles of increasing MNase cleavage of chromatin from Rrn3 wild type and conditional (floxed) MEFs after 72h of treatment with 4-HT. The data in D) and E) was obtained using the 47S hybridization probe shown in C), and the positions of mono- (1), di- (2), etc nucleosomes are indicated. More complete datasets of the MNase analyses are given in S5 Fig.

Chromatin accessibility to psoralen cross-linking has long been used to differentiate active and inactive rRNA genes, a technique believed to be based on the absence or presence of nucleosomes within the 47S transcribed region [53]. The psoralen technique revealed that in the p53^-/-^ MEFs 64 ± 4% of rDNA migrated in the low mobility (psoralen hyper-accessible) active (a) fraction (Fig 4A and 4B). Suppression of RPI transcription by 4-HT induced inactivation of the UBF gene in the *Ubf*^*fl/fl*^*/ER-Cre*^*+/+*^*/p53*^*-/-*^ MEFs eliminated this low mobility band and enhanced the higher mobility band of the inactive genes (Fig 4A), consistent with our previous data [35, 54]. In contrast, suppression of transcription by inactivation of the Rrn3 gene in the *Rrn3*^*fl/fl*^*/ER-Cre*^*+/+*^*/p53*^*-/-*^ MEFs did not affect the psoralen crosslinking pattern (Fig 4B). Thus, the enhanced psoralen accessibility of the active rRNA genes was independent of RPI recruitment or active transcription, and corresponded with the binding of UBF. This is in agreement with data from yeast where the probable UBF ortholog Hmo1 is also sufficient for enhanced psoralen accessibility [55]. It is also an important point when interpreting previous data, since psoralen accessibility can no longer be used as an indicator of active RPI transcription. As will be argued below it is more likely an indicator of the absence of core histones, as was originally suggested [53], and their replacement by UBF.

We further asked if loss of UBF also led to the reformation of nucleosomal chromatin on the previously active rRNA genes. As expected, the rDNA IGS in both conditional and wild type MEFs displayed a “ladder” of Micrococcal Nuclease (MNase) inter-nucleosomal cleavage characteristic of nucleosomal chromatin (S5B and S5C Fig). In contrast, the 47S region displayed a near continuum of MNase cleavage typical of nucleosome-free and actively transcribed DNA. Loss of UBF in *Ubf*^*fl/fl*^*/ER-Cre*^*+/+*^*/p53*^*-/-*^ MEFs (72h post 4-HT) led to the establishment of a nucleosomal cleavage ladder in the 47S region, which now resembled the IGS (Figs 4D and S5B). However, loss of Rrn3 (*Rrn3*^*fl/fl*^*/ER-Cre*^*+/+*^*/p53*^*-/-*^ MEFs, 72h post 4-HT) did not have this effect, the 47S region remaining non-nucleosomal (Figs 4E and S5C). Taken together with the psoralen accessibility analysis (and DNase-Seq, see below), these data showed that UBF was sufficient to exclude nucleosomes from the 47S region of the rDNA. We previously argued that the “Enhancesome” UBF-DNA nucleoprotein structure is incompatible with nucleosomes [56]. However, as discussed above, the low binding constant and the high *in vivo* off-rate both argue that UBF alone would not be able to prevent the incursion of nucleosomes. Rather, the data, suggest the existence of a specific, transcription-independent mechanism for UBF deposition, one perhaps involving chromatin modifying and remodelling complexes [57–59].

### Rrn3-loss causes partial rDNA chromatin collapse but does not disrupt the nucleolus

**Fig 5:**
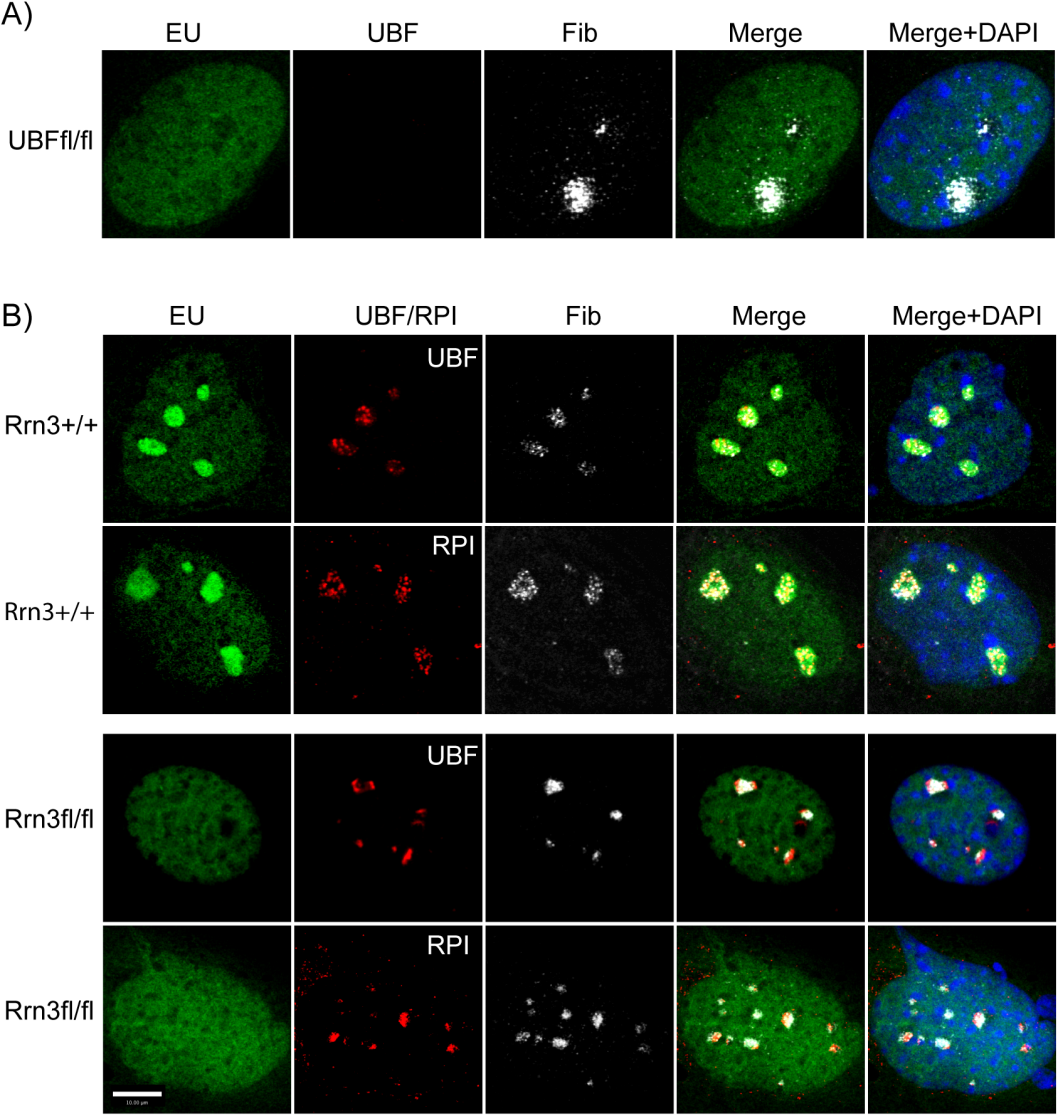
Comparative nucleolar structures before and after UBF or Rrn3 gene inactivation. A) In situ RNA labeling (EU) and UBF and fibrillarin (Fib) staining of *Ubf*^*fl/fl*^*/ER-Cre*^*+/+*^*/p53*^*-/-*^ MEFs 72h post 4-HT treatment. B) In situ RNA labeling (EU), and fibrillarin (Fib) and UBF or RPI staining of *Rrn3*^*wt/wt*^*/ER-Cre*^*+/+*^*/p53*^*-/-*^ and *Rrn3*^*fl/fl*^*/ER-Cre*^*+/+*^*/p53*^*-/-*^ MEFs 72h post 4-HT treatment. Cultures were also counterstained with DAPI.

We previously demonstrated that deletion of the UBF gene led to the complete disassembly of the nucleolus [35, 60]. In the absence of UBF, the RPI transcription machinery and at least one early rRNA processing factor were shown to form a compact somatic nucleolar body (e.g. see Fig 5A), and the rDNA loci shown to scattered throughout the nucleus, suggesting that they collapsed back onto their chromosomal loci, see [35]. When we performed a similar analysis after Rrn3 deletion, we found quite a different nucleolar behavior (Fig 5B). Before deletion, UBF and fibrillarin displayed the expected speckled nucleolar pattern that coincided with the sites of rRNA synthesis (EU incorporation). After Rrn3 deletion, rRNA synthesis was ablated (loss of EU) and UBF and RPI both collapsed into denser, more discrete foci. But, these foci remained immediately proximal to fibrillarin foci and their spatial distribution and number were consistent with the prior sites of active nucleoli. Since under these conditions the rRNA genes remained bound by UBF and SL1 (TAF1s/TBP) (Fig 2), nucleosome-free (Fig 4) and therefore potentially active, the data suggested that the rDNA remained associated with remnant nucleolar structures, and had simply contracted to the heterochromatic edges of the nucleoli, much as seen for transcription inhibition by Actinomycin D [61].

### UBF may replace core histones on the active rDNA

**Fig 6:**
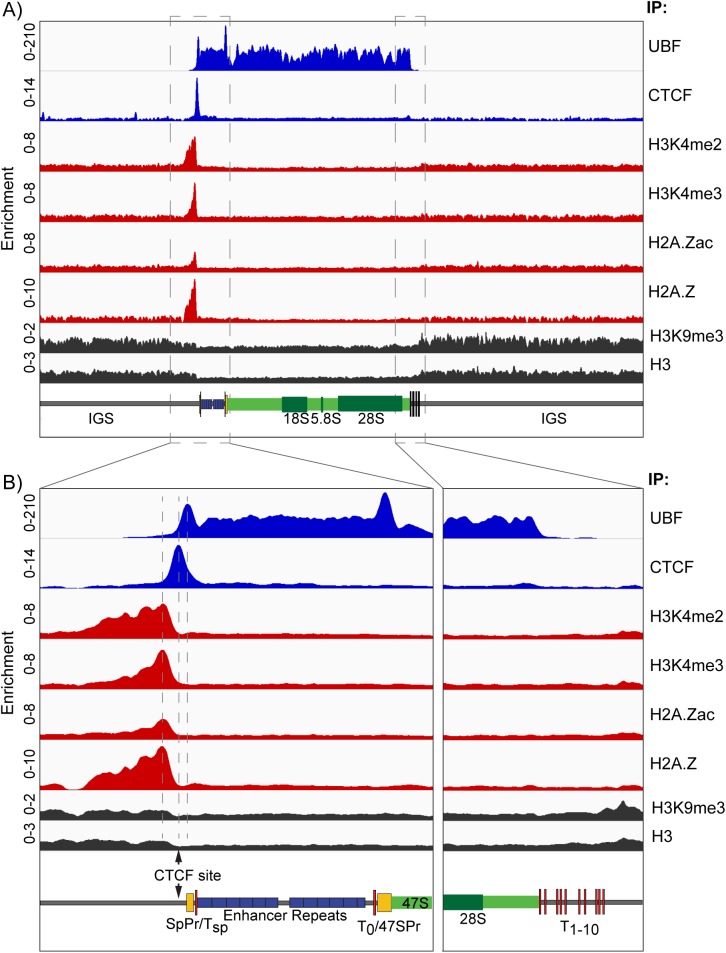
The UBF binding domain is delineated by an Enhancer Boundary Complex. A) The ChIP enrichment profile maps for UBF, CTCF, H3K4me2, H3K4me3, H2A.Zac, H2A.Z, H3K9me3 and H3 across the full rDNA repeat unit. B) Higher resolution maps for the upstream Enhancer and Promoter elements and the 47S termination site (boxed in A). The position of the predicted CTCF binding site [67] is indicated. As in Fig 1, a scale map of the rDNA sequence elements is given below each panel, and ChIP enrichments for each factor are given as; ChIP-Seq RPM/Input DNA RPM.

While our data had suggested that the rDNA IGS was nucleosomal, the 47S region of active genes lacked a nucleosomal cleavage pattern, suggesting a highly disorganized chromatin structure (Figs 4D & 4E and S5B & S5C). However, it was estimated from psoralen crosslinking that 64 ± 4% of the rDNA in MEFs was active and 35 ± 4% inactive (Fig 4A & 4B). The inactive repeats might be expected to be nucleosomal throughout, much as we observed after UBF loss (Fig 4D). Consistent with this, histone H3 and the heterochromatic marker H3K9me3 were detected throughout the rDNA, but both were reduced to roughly 40% of the IGS value over the Enhancer and 47S gene regions (Fig 6A). The data therefore suggested that H3 and H3K9me3 were predominantly absent from the Enhancer and 47S gene regions of active genes, but occupied the full rDNA repeat of inactive genes as well as the IGS of the active genes. This suggested that the gene bodies of the active rDNA may be histone-free and instead occupied by UBF. This situation would be reminiscent of the chromatin status of the yeast rDNA, where Hmo1 replaces the histones on active genes [62].

### The domain of UBF recruitment is delineated by a unique upstream Enhancer Boundary Complex

Despite around 60% of the rRNA genes being active in the MEFs, our ChIP-Seq normalization procedure revealed a complete lack of active or potentially active chromatin marks across the Spacer and 47S promoters, the Enhancer repeats, the 47S gene body and all but a very small region of the IGS (Fig 6A). This was very surprising considering the extensive literature suggesting active histone modifications and nucleosome sliding at the 47S promoter regulate its activity, reviewed in [63–65]. The only exception was a site immediately 5’ of the Spacer Promoter at which we observed strong and overlapping occupancy of H3K4me2, H3K4me3, H2A.Z and H2A.Zac (K4ac/K7ac/K11ac) [66]. Our realignment of publicly available ChIP-Seq data also revealed unique and overlapping peaks of H3K4me1/me2/me3, H3K36me3, H3K9ac and H3K27ac (ENCODE GSE32218) at the same site in mouse embryonic stem cells (mESC), (S6 Fig), and a similar observation for H3K4me2/me3 was made by Zentner et al. [34] by realignment of public data sets (GSE11172, GSE8024) also from mESCs. This concentration of activating marks was flanked immediately 3’ by a unique site occupied by the genome architecture and boundary CCCTC-binding factor CTCF [67, 68], which in turn immediately flanked the upstream boundary of UBF binding (Fig 6A & 6B). Thus, the active Enhancer and 47S transcribed regions of the rDNA repeats were flanked on their 5’ side by a unique chromatin boundary complex that contained multiple marks of active and poised chromatin. The lack of active chromatin marks within the functional rRNA gene is consistent with a near total lack of core histone in this UBF-bound region. As the unique exception to this, the Enhancer Boundary Complex appeared likely to be a key structure in the control of gene activity.

### The Enhancer Boundary Complex is maintained in the absence of UBF

**Fig 7:**
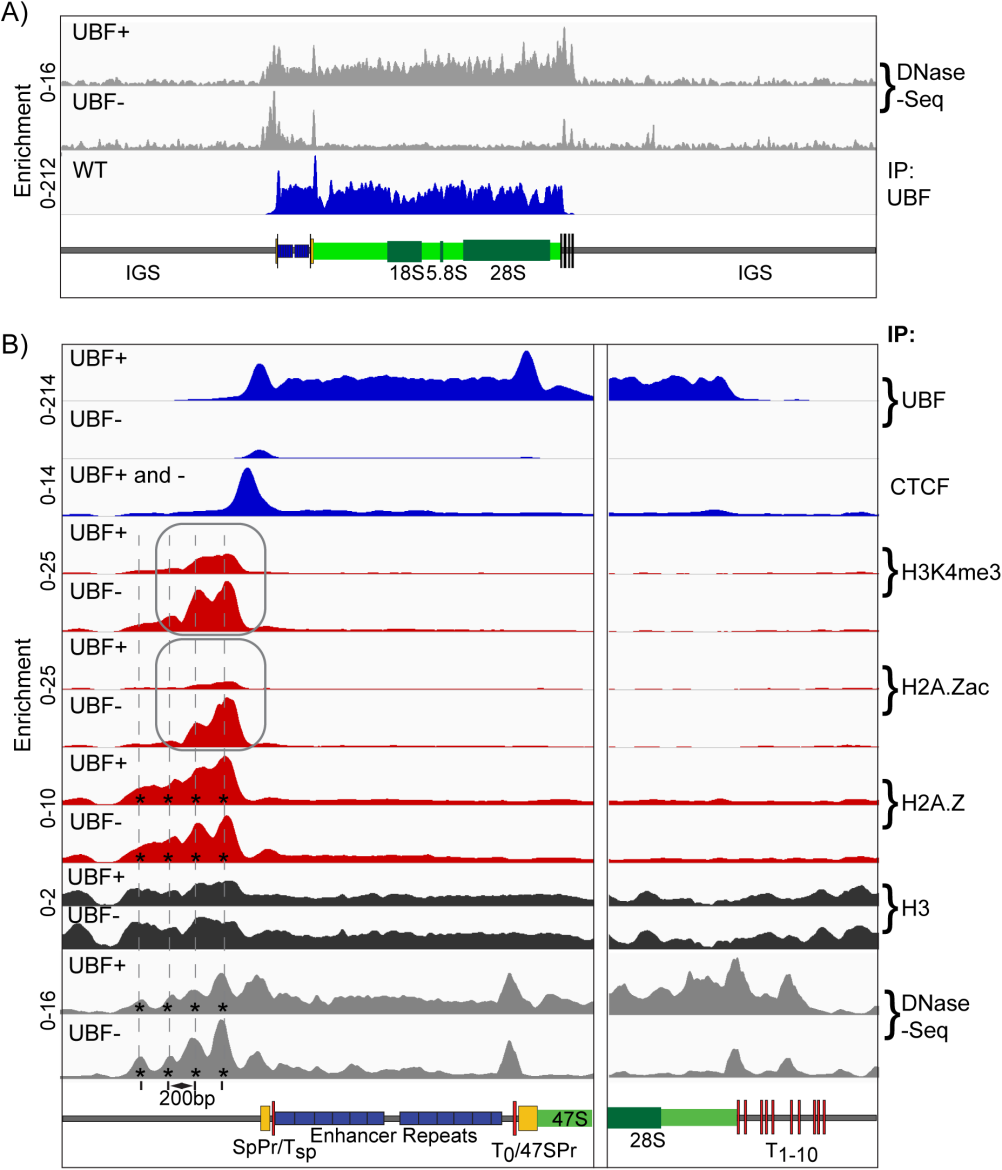
The Enhancer Boundary Complex exists independently of UBF and rDNA activity. A) DNase-Seq analysis of *Ubf*^*wt/wt*^*/ER-Cre*^*+/+*^*/p53*^*-/-*^ (UBF+) and *Ubf*^*fl/fl*^*/ER-Cre*^*+/+*^*/p53*^*-/-*^ (UBF-) MEFs 72h post 4-HT treatment across the full rDNA repeat unit as compared to the UBF ChIP-Seq profile of *Ubf*^*wt/wt*^*/ER-Cre*^*+/+*^*/p53*^*-/-*^ (WT) MEFs also 72h post 4-HT treatment. B) Higher resolution ChIP enrichment profile maps for UBF, CTCF, H3K4me3, H2A.Zac, H2A.Z and H3 across the upstream Enhancer and Promoter elements and the downstream 47S termination site of *Ubf*^*fl/fl*^*/ER-Cre*^*+/+*^*/p53*^*-/-*^ MEFs either untreated (UBF+) or 72h post 4-HT (UBF-). Binding of CTCF (UBF+ and -) was unaffected by UBF loss. Higher resolution DNase-Seq profiles are also given in panel B) as are the positions of phased nucleosomes (*). Scale maps of the rDNA sequence elements are provided below each panel. Enrichments for each track are given as; Sample DNA RPM/Input DNA RPM.

We found that the Enhancer and 47S regions of the rDNA were highly sensitive to DNase I, again suggesting a very open structure consistent with UBF binding. This hypersensitivity was eliminated when UBF was deleted (Fig 7A), and corresponded with the loss of psoralen accessibility and the formation of a nucleosome ladder (Fig 4). In contrast, the site occupied by the Enhancer Boundary Complex remained DNase hypersensitive even after UBF deletion. We therefore asked if the components of this Complex were also maintained.

**Fig 8:**
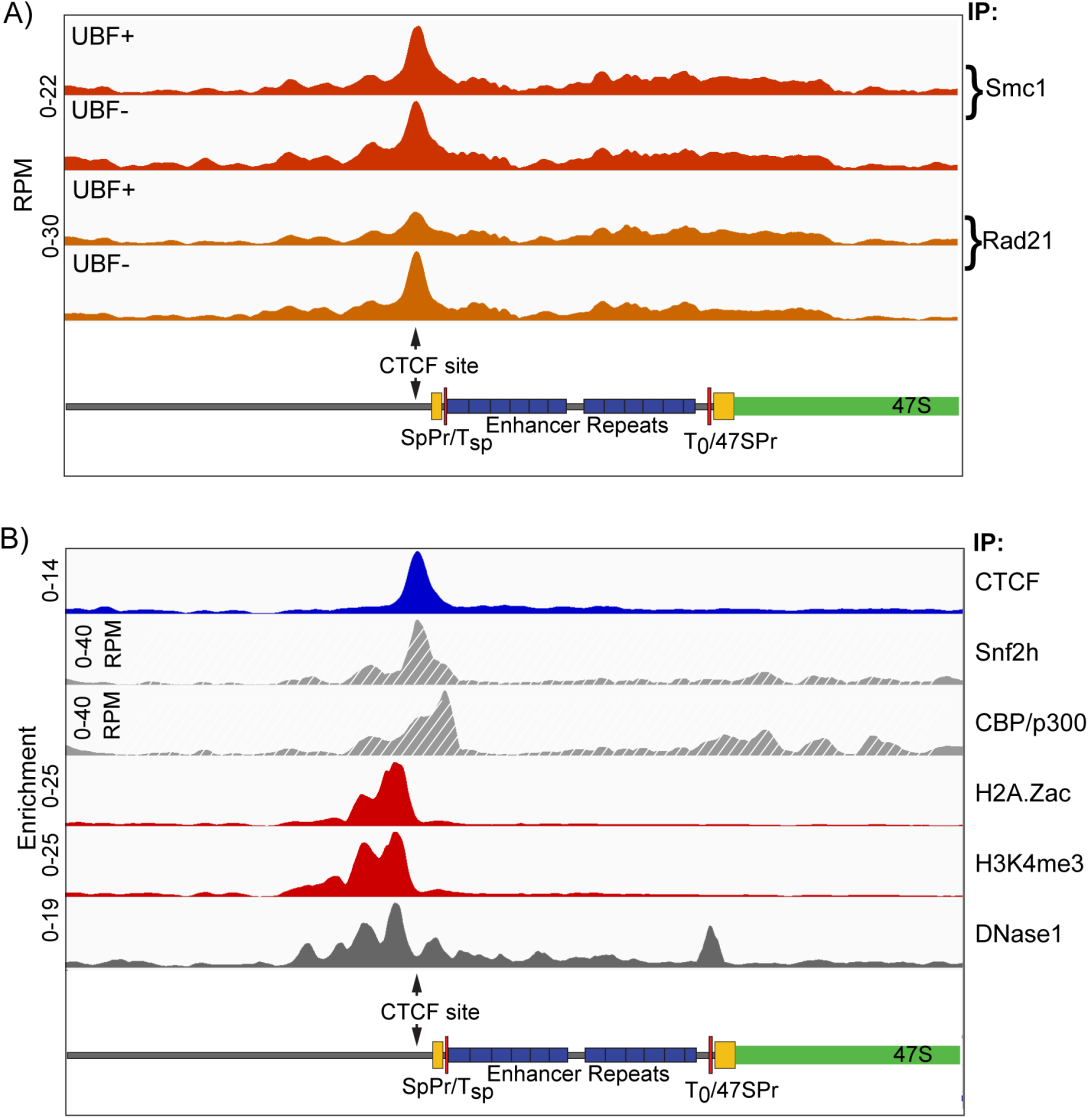
Cohesin subunits and chromatin remodelers also map to the Enhancer Boundary. A) Distribution of Cohesin subunits Smc1 and Rad21 across the rDNA repeat unit of *Ubf*^*fl/fl*^*/ER-Cre*^*+/+*^*/p53*^*-/-*^ MEFs either untreated (UBF+) or 72h post 4-HT (UBF-). B) Comparative distribution of CTCF, H2A.Zac, H3K4me3 and DNase1 cleavage across the mouse rDNA from the present study and Snf2h (GSE53583) [91] and CPB/p300 (GSE54453) [92]. Scale maps of the rDNA sequence elements are provided below each panel. Enrichments for each track are given either as; Sample DNA RPM/Input DNA RPM, or directly in RPM as indicated.

CTCF binding to the unique Spacer Promoter adjacent site was predominantly maintained or somewhat enhanced after Rrn3 or UBF loss and H2A.Z binding immediately upstream of this site was unaffected by the loss of UBF (S7 Fig and Fig 7B). Surprisingly, the H3K4me3 active chromatin mark was not only maintained, but was enhanced by the loss of either Rrn3 or UBF (Fig 7B and S7 Fig), and H2A.Zac was also significantly enhanced after UBF loss (Fig 7). These active chromatin marks followed a 200bp repeat pattern that was reiterated in the DNase-Seq pattern of fragment release, suggesting that they lay within three or four phased nucleosomes immediately adjacent to the CTCF site (indicated by asterisks in Fig 7B, see also Fig 8B). These phased nucleosomes clearly displayed reducing degrees of H3K4me3 and H2A.Zac modification with increasing distance upstream from CTCF and from the 5’ UBF boundary.

### Cohesin is also recruited to the Enhancer Boundary Complex

CTCF is known to recruit the Cohesin complex via it C-terminal domain, and indeed in many cases Cohesin is required for the maintenance of CTCF binding [68]. Our ChIP-Seq analyses revealed the presence of the Cohesin subunits Smc1 and Rad21 exactly overlapping the peak of CTCF binding (Fig 8A). As for CTCF and the active histone marks, Cohesin recruitment was in predominantly independent of UBF and hence also of gene activity. Given the stability of not only CTCF and Cohesin at the Enhancer boundary through many hours of complete gene inactivation, but also of the maintenance of the active chromatin marks despite the reestablishment of surrounding repressive nucleosomal chromatin (see again Figs 4 and 7), we suggest that the formation of the Enhancer Boundary Complex must be an early event in rDNA activation. In support of this, realignment of public ChIP-Seq data further revealed that the Enhancer Boundary is the major site of binding for the central SWI/SNF chromatin remodeller subunit Snf2h/SMARC5 and the histone acetyltransferase (HAT) CPB/p300, known to directly bind and acetylate UBF [57] (Fig 8B). Thus, the assembly of the Enhancer Boundary Complex is likely to be the dominant determinant of rDNA activity.

## Discussion

Using a novel ChIP-Seq normalization protocol we have been able to generate *in vivo* high-resolution maps of RPI transcription factor and chromatin interactions across the full mouse rDNA repeat in both the presence and absence of the two key regulatory factors Rrn3 and UBF. The level of mapping detail provides an in-depth understanding of active rDNA chromatin, Spacer Promoter function, RPI transcription and termination, and the interdependence of basal transcription factors. The data further reveal the existence of a unique Enhancer Boundary Complex that marks the rDNA repeats even in the complete absence of active transcription and the basal factors, and suggest that this complex could be a key entry site for factors that drive and/or maintain rDNA activation.

**Fig 9:**
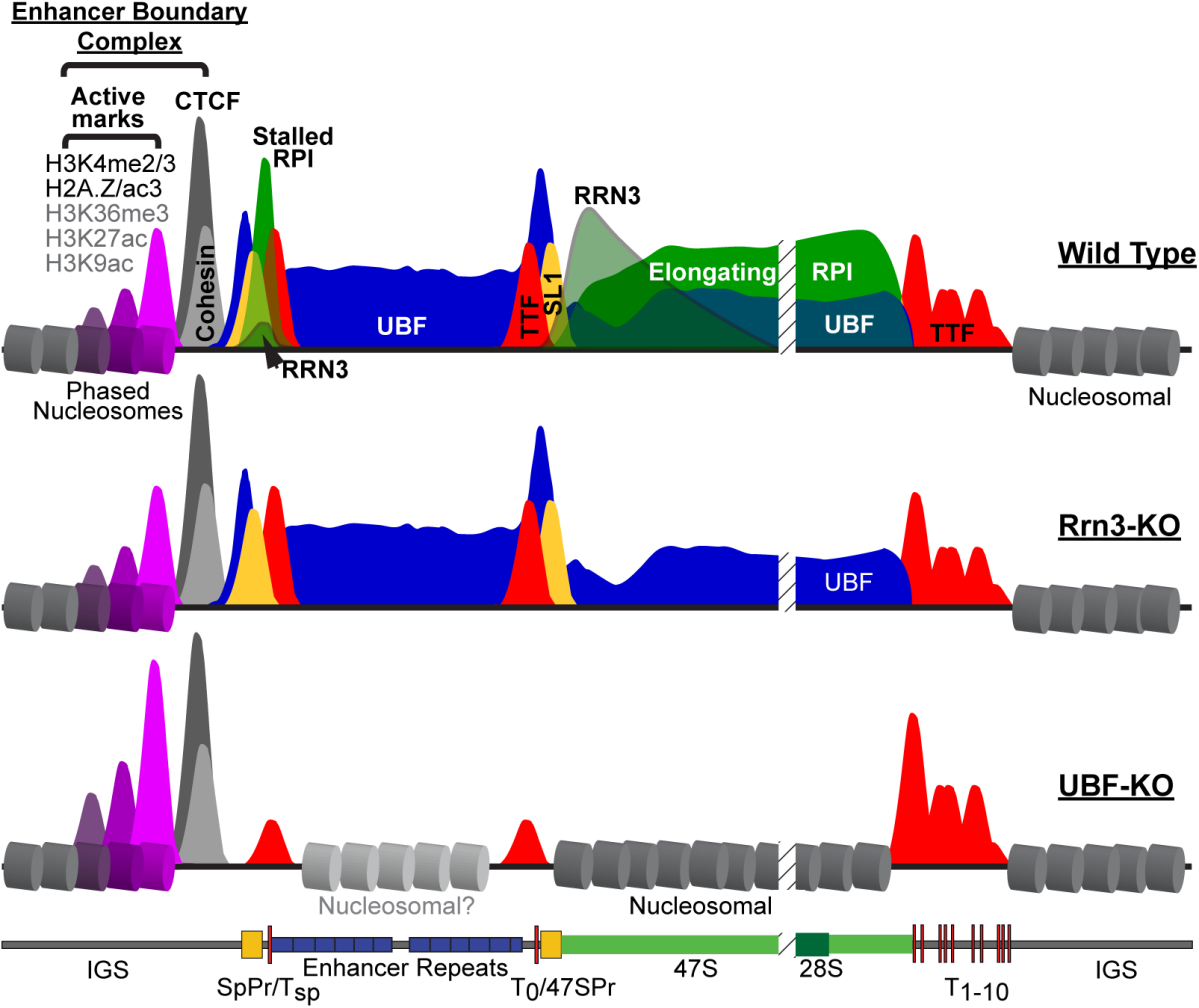
Diagrammatic summary of factor binding and chromatin structure across the active mouse rDNA repeat (Wild Type) and after deletion of Rrn3 (Rrn3-KO) or UBF (UBF-KO). UBF is indicated in blue, RPI in green, Rrn3 in grey/green, TTF1 in red, SL1 in yellow, CTCF in dark grey and Cohesin in light grey. The CTCF adjacent phased nucleosomes are indicated in magenta and their degree of modification indicated by the height of corresponding peaks.

We found that UBF is present throughout the 47S gene body and across the Spacer Promoter and Enhancer repeats of active genes. The UBF binding domain is immediately flanked on the upstream side by a unique Enhancer Boundary Complex of CTCF and Cohesin and then by nucleosomal chromatin. Three or four phased nucleosomes lie adjacent to CTCF/Cohesin and form the sole site in the rDNA marked by active chromatin modifications (H3K4me2/3, H2A.Z, H2A.Zac and probably H3K9ac, H3K27ac and H3K36me3), the rest of the rDNA repeat including both RPI promoters and the UBF-bound domain completely lacking active histone marks. On the downstream side, the UBF domain stops abruptly at the first two TTF1-bound termination sites, and these are followed further downstream by nucleosomal chromatin. These data are summarized in Fig 9, and we will return to consider their importance after first discussing the mapping of the RPI transcription machinery and the effects of Rrn3 and UBF deletion on rDNA activity and chromatin.

Clear peaks of UBF binding are found overlapping the mapped 47S and Spacer Promoter sequences, and correspond to sites bound by the SL1 preinitiation complex. RPI maps throughout the 47S gene body, but association of Rrn3 with RPI reduces exponentially starting from the site of initiation, and is consistent with stochastic release of Rrn3 from the elongation complex. The RPI termination factor TTF1 maps to the T_1-10_ termination sites with a preference for the T_1_ and T_2_ sites, but also maps to the predicted 47S Promoter and Spacer Promoter adjacent T_0_ and T_sp_ sites. Interaction of RPI with the 47S gene body ends abruptly at the T_1-10_ cluster of sites, and no RPI (≤ 2%) is detected further downstream. This suggests that TTF1 bound at T_1-10_ is sufficient to arrest the RPI elongation complex and promote its disruption, in agreement with recent findings in yeast [69]. RPI elongation complexes display a very uniform profile across the 47S gene body (Fig 1A & 1B, and “Elongating RPI” in Fig 9), excluding significant sequence-specific pausing. In contrast, RPI elongation complexes initiating at the Spacer Promoter are arrested at the adjacent Tsp site, most probably by TTF1 (Figs 1D and 9). It has been proposed that long non-coding RNAs (lncRNAs) initiated from the Spacer Promoter are processed to generate the pRNA required for rDNA silencing [16, 17, 70, 71]. Thus, TTF1 bound at the Tsp site controls synthesis of the pRNA precursor and hence the potential for rDNA silencing. Consistent with this, TTF1 abundance and subnuclear localization were previously shown to regulate rDNA activity [52, 72].

Normalized ChIP-Seq in conditional MEFs showed loss of Rrn3 blocked RPI initiation at both 47S and Spacer Promoters and prevented synthesis of the 47S rRNA. It did not, however, have any effect on SL1 preinitiation complex formation at either promoter. Further, Rrn3-loss did not affect the profile of UBF binding, nor psoralen accessibility of the rDNA. Thus, in agreement with data from yeast [55], RPI transcription appears not to be required to maintain UBF binding and the psoralen accessible status of the rDNA in MEFs. The data also infer that elongating RPI must be able to traverse the UBF-DNA complex without displacing it, and is consistent with the role of ERK and CBP modification of UBF in regulating RPI passage [18, 23, 57, 73]. As spin-off from our Rrn3-inactivation study, we were able to allay doubts as to the essential nature of the mouse *Rrn3(Tif1a)* gene (S2 Fig).

In contrast to Rrn3-loss, deletion of UBF eliminated not only all interaction of RPI and Rrn3 with the rDNA, but also SL1 preinitiation complex formation at both 47S and Spacer promoters. Thus, as predicted from the earliest in vitro studies [74], SL1 recruitment depends on the presence of UBF. However, our data do not speak to any cooperativity between UBF and SL1 recruitment as has also been suggested [75], and this must await the results of ongoing studies of SL1 inactivation. UBF, but not Rrn3 deletion, reduced binding of TTF1 to its promoter associated sites and increased binding at the termination sites. Further, both Rrn3 and UBF loss affected the low level TTF1 interaction throughout the 47S gene body, suggesting a dynamic RPI-dependent cycling between these sites.

Psoralen accessibility is used to distinguish active and inactive rDNA repeats [53], and has long been assumed to detect active RPI transcription. However, we found that deletion of Rrn3 had no effect on psoralen accessibility, while UBF deletion eliminated it. Thus, psoralen accessibility is clearly a property of UBF bound rDNA and not an indicator of active RPI transcription. Consistent with this, the UBF domain of the rDNA was DNase hypersensitive and lacked nucleosomal chromatin, while deletion of UBF led to the reformation of nucleosomes and the loss of this DNase hypersensitivity. Our data also suggest that not only nucleosomes, but also histones may be absent from the regions bound by UBF, potentially explaining the complete lack of activating histone modifications over the rRNA gene unit.

Strikingly, the Enhancer Boundary Complex, consisting of CTCF, Cohesin and the adjacent phased nucleosomes, was not perturbed by UBF deletion and ChIP-QPCR analyses showed this was also the case for Rrn3 deletion. (summarized in Fig 9). This boundary complex was all the more remarkable for being the sole site of activating histone modifications (H3K4me2 and H3K4me3, H2A.Z and H2A.Zac, and probably H3K36me3, H3K9ac and H3K27ac) in the whole rDNA repeat, and showed itself to be highly stable, remaining many hours after UBF deletion and the release of all RPI transcription factors. Indeed, the associated H3K4me3 and H2A.Zac modifications were even enhanced by UBF deletion. The Enhancer Boundary Complex, therefore, represents a stable marker of the potentially active or poised rDNA fraction.

The Enhancer Boundary Complex also displays a striking asymmetry, on its upstream side CTCF being flanked by phased nucleosomes containing the near full range of activating histone modifications and the H2A.Z histone variant, and on its downstream side by an a SL1/UBF PIC and a transcriptionally engaged RPI complex. CTCF binding sites are in themselves asymmetric, and this is believed to enable 180deg. DNA looping-out via CTCF interactions between distant palindromic sites [68] or the formation of 360deg. circular loops by interactions between tandemly oriented sites, e.g. see [76]. It was previously noted that the unique CTCF site in the mouse rDNA is oriented away from the gene body [67]. Thus, CTCF interactions between adjacent gene repeats would be unlikely to induce 180deg. looping-out unless they involved heterologous interactions, e.g. with TTF1 [42]. They would, however, permit 360deg. circular looping analogous to that suggested for the SL1 complex [77], though at the scale of the whole rDNA repeat. Alternatively, it was noted that in human the tandem repeated rDNA loci are interspersed with inverted, often incomplete, rDNA units [78]. If such aberrant repeats are present in mouse, by presenting inverted CTCF sites, they might induce the formation of 180deg. loops containing multiple rDNA units. In any of these scenarios, the role of Cohesin at the site of the Enhancer Boundary Complex would be expected to stabilize looping via this site [68, 76], and could play an important role in defining active rDNA arrays, and/or controlling inter-repeat recombination. Indeed, data from yeast and human has already suggested Cohesin plays an important role in the activity and stability of the rDNA arrays [79].

The observation that no active histone marks occur within the mouse rRNA gene body, the RPI promoters or the Enhancers conflicts with several studies suggesting an importance of histone modification, particularly at the 47S Promoter, in activating gene transcription, see [63] for review. It is, however, difficult to imagine how, as has been suggested, nucleosomes could co-exist with the preinitiation complex and UBF at the 47S promoter of actively transcribed genes. Indeed, the data from yeast has shown that little or no histone can be detected at the 35S rRNA promoter [62] and so is in full agreement with our findings. Further, public ChIP-Seq data also failed to detect significant active histone modifications over the functional rRNA gene. Together the data suggest that active histone modifications could only play a transient role, perhaps during the displacement nucleosomes and their replacement by UBF. As we have shown, once it is recruited UBF remains stably bound across the functional rRNA gene even in the absence of active transcription, leaving little chance for histone modifications to play a regulatory role.

We previously showed that the rDNA exists in not just two, but three distinct states, heterochromatin and CpG methylated, a poised state defined at the time by low psoralen accessibility but completely lacking DNA methylation and an actively transcribed state defined by high psoralen accessibility [80, 81]. Since we now show that the psoralen assay classifies active and inactive genes based solely on UBF-binding, we can suggest a refinement to our definition of the poised rDNA state as one that is nucleosomal throughout, but is marked by the Enhancer Boundary Complex. Preliminary data suggest that this boundary complex is also present on the human rDNA. We suggest that the Enhancer Boundary Complex is the initial site from which rDNA activity is induced. This is supported by the presence of the ATPase chromatin remodeling factor Snf2h (SMARCA5) and probably the acetyltransferase CBP/p300 at this boundary. It is also in agreement with data showing that loss of CTCF reduces the recruitment of UBF throughout the gene body as well as the recruitment of RPI to the Spacer Promoter [67]. These authors also showed that binding of CTCF to the Spacer Promoter adjacent site occurs predominantly on the unmethylated and so potentially active rDNA fraction. Taken together, the data strongly suggest a model for the active rDNA repeats in which UBF replaces histone chromatin from the Spacer Promoter through to the TTF1 bound T_1-10_ termination sites (Fig 9), and that this situation is established or maintained by chromatin modifying activities emanating from the upstream Enhancer Boundary Complex.

## Materials and methods

### Isolation and culturing of MEFs

The generation of conditional *Ubf*^*fl/fl*^*ER-Cre*^*+/+*^ and control mouse lines was previously described (Hamdane et al. 2014). *Ubf*^*fl/fl*^*/ER-Cre*^*+/+*^*/p53*^*-/-*^ and *Ubf*^*+/+*^*/ER-Cre*^*+/+*^*/p53*^*-/-*^ isogenic mice were generated by introducing the p53-null allele from strain *129-Trp53*^*tm1Tyj*^*/J* (Jackson Laboratory #002080) [82]. To generate *Rrn3*^*+/-*^, *Rrn3*^*fl/fl*^*/ER-Cre*^*+/+*^*/p53*^*-/-*^ and isogenic *Rrn3*^*+/+*^*ER-Cre*^*+/+*^*/p53*^*-/-*^ mice, the following mouse strains from Jackson Laboratory were used; B6.Cg-Tg(Sox2-cre)1Amc/J (#008454), B6;129-*Gt(ROSA)26Sor*^*tm1(cre/ERT)Nat*^/J (#004847) and B6.Cg-Tg(UBC-cre/ERT2)1Ejb/2J (#008085). C57BL/6NCrl wild-type mice for backcrossing were from Charles River. Primary mouse embryonic fibroblasts (MEFs) were generated from E14.5 *Ubf*^*fl/fl*^*/ER-Cre*^*+/+*^*/p53*^*-/-*^, *Rrn3*^*fl/fl*^*/ER-Cre*^*+/+*^*/p53*^*-/-*^, and equivalent *Ubf*^*+/+*^ and *Rrn3*^*+/+*^ embryos as previously described [35, 83]. MEFs were cultured in Dulbecco’s modified Eagle medium (DMEM)-high glucose (Life Technologies), supplemented with 10% fetal bovine serum (Wisent), L-glutamine (Life Technologies) and Antibiotic/Antimycotic (Wisent).

### Embryo collection and genotyping

Heterozygous *Rrn3*^*+/-*^ mice were inter-crossed and embryos isolated from pregnant females at 3.5, 6.5, 7.5, 8.5, 9.5 and 10.5dpc. DNA from 3.5 dpc embryos was amplified using the REPLI-g Mini kit (QIAGEN). Genotyping on DNA from all embryo stages was performed by PCR using the same primers as for cell lines, see below.

### Inactivation of *Ubf* or of *Rrn3* in cell culture, and analysis of genotype, rRNA synthesis and proteins

As previously described (Hamdane et al. 2014), cells were initially plated in 6 cm petri dishes (0.8x10^6^ cells each) and cultured for 18 hours in DMEM, high glucose, 10% fetal bovine serum. To initiate *Ubf* inactivation, 4-hydroxytamoxifen (4-HT) was added to both *Ubf*^*fl/fl*^ and *Ubf*^*+/+*^ cell cultures to a final concentration of 50nM (the 0h time point for analyses), and after 4 hr incubation the medium replaced with fresh medium without 4-HT [35]. Cultures were then subjected to analysis at various subsequent time points. In the case of *Rrn3*, cells were treated with 50nM 4-HT for 4h, passaged and diluted 1:2 and retreated 5h later with 50nM 4-HT for 15h, and this treatment protocol immediately repeated. Finally, the cells were replated at 80% confluency (the 0h time point for analyses), treated with 50nM 4-HT for 15h, and the medium replaced with fresh medium without 4-HT, before analysis at various subsequent time points. Comparative colony forming assays showed that generally less than 0.01% of *Ubf*^*fl/fl*^ cells formed colonies after 4-HT treatment, while a few percent of *Rrn3*^*fl/fl*^ cells were still able to form colonies and hence still carried a functional *Rrn3* allele. Analyses systematically included genotyping and UBF and Rrn3 protein level determination. Cells were genotyped by PCR using the following primers, for *Ubf*: A; 5’TGATCCCTCCCTTTCTGATG, B; 5’TGGGGATAGGCCTTAGAGAGA, C; 5’CACGGGAAAACAAGGTCACT and for *Rrn3*: A; 5'-GATCTTAATGGAGGGCAGCA, B; 5’-TGGATCCTGCAACTTTTTCC, C; 5’ TCCCAACCCTGACCTATCAC. To determine UBF or Rrn3 protein levels, cells were washed with cold phosphate buffered saline (PBS), scraped into PBS, centrifuged 2 min at 2000 r.p.m., then resuspended in SDS–polyacrylamide gel electrophoresis (SDS-PAGE) [84] loading buffer. After fractionation on 8% SDS-PAGE, cell extracts were analysed by standard Western blotting procedures. Metabolic labelling of RNA was carried out immediately before cell harvesting by the addition of 10 μCi [^3^H]-uridine (PerkinElmer) to the culture medium and incubation for a further 3h. RNA was extracted using Trizol (Life Technologies) according to the manufacturer’s protocol, analyzed by gel electrophoresis and fluoroimaging (Enhance, PerkinElmer) and RNA species quantitated by scintillation counting as previously described [18, 48, 73].

### Colony forming assays

*Rrn3*^*fl/fl*^*-*, and *Ubf*^*fl/fl*^*/ER-Cre*^*+/+*^*/p53*^*-/-*^ and matched *Rrn3*^*+/+*^*-*, and *Ubf*^*+/+*^*/ER-Cre*^*+/+*^*/p53*^*-/-*^ MEFs were treated with 4-HT as described above, and 48h later each culture was replated in duplicate at dilutions of 10 000, 50 000, 100 000 cells per 60 mm petri. 144h post 4-HT treatment, petri dishes were fixed for 5 mins with 4% paraformaldehyde/PBS and stained with 0.05% crystal violet in distilled water (filtered) for 30 mins. Dishes were then rinsed 3 times with water and left inverted to dry. Crystal violet staining was quantified by adding 1ml of methanol to each dish to solubilize the dye, the methanol recovered and its optical density at 540nm determined after appropriate dilution.

### Antibodies for western blot, immunofluorescence and ChIP

Rabbit polyclonal antibodies against mouse UBF, RPI large subunit (RPA194/Polr1A), TTF1, TAF1B (TAF68) and RRN3 were generated in the laboratory. The UBF, RPI and TTF1 antibodies have been previously described [52]. Rabbit antisera were raised against TAF68 aa 54–175 and RRN3 aa 464–656, expressed in E. coli and peptides were purified using the guanidinium chloride-urea denaturation method. Rabbit antibody against TAF1C (TAF95) was a gift from Ingrid Grummt. Anti-H2A.Z, anti-H2A.Zac and ant-H3K4me2 were gifts from Colyn Crane-Robinson. All other antibodies were obtained commercially; TBP (#ab818 Abcam), anti-Tubulin (#T5168 Sigma), anti-Fibrillarin (#905001 BioLegend), anti-CTCF (#07–729 Millipore), anti-SMC1 (#A300-055A Bethyl), anti-Rad21 (#ab992 Abcam) anti-H3K4me3 (#ab1012 Abcam), anti-H2A.Z (#ab4174 Abcam), anti-H3K9me3 (#17–625 Millipore), anti-H3 (#ab1791 Abcam).

### Chromatin immunoprecipitation (ChIP)

Cells were fixed with 1% formaldehyde for 8 min at room temperature. Nuclei were isolated using Lysis Buffer (10 mM Tris pH 7.5, 10 mM NaCl, 3 mM MgCl2, 0.5% NP-40), transferred to Sonication Buffer (50 mM Tris-HCl pH 7.5, 150 mM NaCl, 2 mM EGTA, 4 mM EDTA, 0.1% SDS, 1% Triton X-100, 1% NP-40) and sonicated (Bioruptor, Diagenode) for 30 cycles of 30 sec on / 30 sec off at high intensity. Each immunoprecipitation (IP) was carried out on the equivalent of 50 x 10^6^ cells in IP Buffer (150 mM NaCl, 50 mM Tris-HCl pH 7.5, 5 mM EDTA, 0.5% NP-40, 1% Triton X-100) overnight at 4°C. The antibody slurry was prepared with 50 μl A-, 50 μl G-Dynabeads and 60 μg.ml^-1^ antibody per IP. Immunoprecipitated chromatin was treated with RNaseA and the DNA isolated using 2% Na SDS and 2mg.ml^-1^ Proteinase-K. Two or more biological replicates were analyzed for each antibody.

### Analysis of ChIP samples by massively parallel sequencing

ChIP DNA samples were quality controlled by qPCR before being sent for library preparation and 50 base single-end sequencing on an Illumina HiSeq 2000 (McGill University and Génome Québec Innovation Centre). For qPCR analysis, reactions (20 μl) were performed in triplicate using 2.5 μl of sample DNA, 20 pmol of each primer, and 10 μl of Quantitect SYBR Green PCR Master Mix (QIAGEN). Forty reaction cycles of 10 s at 95°C and 30 s at 58°C were carried out on a Multiplex 3005 Plus (Stratagene/Agilent). The amplicon coordinates relative to the 47S rRNA initiation site (BK000964v3) were as follows: IGS3, 42646–42903; SpPr, 43089–43253; Tsp, 43267–43421; 47SPr, 45133–40; 47S, 159–320; ETS, 3078–3221; 28S, 10215–10411; T1–3, 13412–13607. Data was analyzed using the MxPro software (Agilent). The relative occupancy of each factor was determined by comparison with a standard curve of amplification efficiency for each amplicon using a range of input DNA amounts and generated in parallel with each qPCR run.

### DNase-Seq

DNase-Seq analysis of MEFs was carried out following the published protocol [85], the only modifications being adjustment of digestion level to obtain the required fragmentation. DNA samples were quality controlled by qPCR before size selection following the published protocol. The resultant DNA was sent for library preparation and single-end sequencing on an Illumina HiSeq 2000 (McGill University and Génome Québec Innovation Centre).

### Analysis of massively parallel sequence data

The data analysis pipeline was developed to take best advantage of the sequencing depth achievable across the rDNA. We noted that sequence coverage was strongly, but reproducibly biased by the underlying DNA sequences regardless of sample type and devised a simple approach to correct for this bias. Briefly, the raw sequence data from experimental and input DNA samples was checked for quality using FastQC version 0.11.4 (Babraham Bioinformatics, S. Andrews). The data was then trimmed using Trimmomatic version 0.33 [86] and the resulting quality filtered files were aligned to the mouse genome version MmGRCm38 to which a single copy of the mouse rDNA repeat sequence (GenBank BK000964v3) was added as an extra chromosome using Bowtie2 [87]. For convenience, the origin of the rDNA repeat was displaced to the EcoRI site at 30,493 such that the pre-rRNA initiation site now fell at nucleotide 14,815. Aligned reads were extended to 100bp (fragment sizes estimated using HOMER fell between 75 and 125bp [88]), the coverage calculated using BEDtools (Quinlan Lab, University of Utah), and smoothed using a window of 25 bp as:

$$J=\frac{1}{25}•\sum_{n+12}^{n-12}j_{n}$$ (1)

where *J* = smoothed base coverage, *j* = aligned coverage and *n* = base position).

The data was then converted to reads per million (RPM) and the sample DNA coverage normalized to the input DNA coverage. The normalized sample data *J*_*norm*_ was calculated for each base position using AWK in Ubuntu as:

Jnorm=JchipJinput (2)

where *J*_*ChIP*_ = smoothed sample DNA coverage, and *J*_*input*_ = smoothed input DNA coverage.

The resulting normalized BED file was converted to BEDgraph format and visualized using IGV (Integrative Genomics Viewer 2.3, Broad Institute).

The ChIP-Seq and DNase-Seq data have been deposited in the ArrayExpress database at EMBL-EBI (www.ebi.ac.uk/arrayexpress) under accession number E-MTAB-5839.

### Psoralen crosslinking accessibility

The psoralen crosslinking accessibility assay was performed on cells grown in 60 mm petri dishes as previously described [53, 89], using the 6.7kb 47SrRNA gene EcoRI fragment (pMr100) [53] and the 6.2 kb EcoRI IGS fragment [90]. The ratio of “active” to “inactive” genes was estimated by analyzing the intensity profile of low and high mobility bands revealed by phospho-imaging on a Fuji FLA-5100 (FUJIFILM Life Science) using a Gaussian peak fit generated with MagicPlotPro (MagicPlot Systems).

### MNase digestion of chromatin

After trypsinizing and washing with PBS the cells were resuspended in buffer A (60 mM KCl, 15 mM NaCl, 15 mM TrisHCl pH 7.6) plus 0.25 M sucrose, 1 mM EDTA, 0.1 mM EGTA, 0.5 mM spermine, 0.15 mM spermidine, 0.1 mM PMSF and protease inhibitors. Triton X100 was added to final concentration 0.25% and the cells homogenized in a Dounce homogenizer using 10 piston strokes and centrifuged 5 min at 2000 rpm. The pellet was washed once by resuspending in buffer A plus 0.34M glucose and then centrifuged for 5 min at 2000 rpm. It was then resuspended in 1 ml buffer A without sucrose, recentrifuged and resuspended in buffer A containing 60 units MNase per 10^7^ cells. After incubating for 2 min on ice, 1 mM CaCl_2_ was added and incubation continued on ice. 200 μl aliquots were taken after 5, 10, 15, 20, and 30 min and the reaction was stopped by adding EDTA to final concentration of 10 mM, SDS to 0.5% and Proteinase K to 1 mg.ml^-1^. After overnight incubation at 55°C, 0.1 mg.ml^-1^ RNase A and 3 units RNase T1 were added and incubated for 1 h at 37°C. This was followed by the addition of 1 mg.ml^-1^ Proteinase K for 2 h at 55°C. The DNA was then extracted with phenol-chloroform, precipitated and dissolved in 200–400 μl TE pH8.3. 10 μl DNA was mixed with 2 x gel loading buffer containing 0.1% SDS and 0.1 mg.ml^-1^ RNase A without dye. After 30 min incubation at 37°C the samples were resolved on 1.5% agarose gel, the gel transferred onto a Biodyne B (Pall) membrane and nucleosome ladders detected by DNA hybridization using the same probes as for the psoralen analysis.

### In situ immunofluorescence, EU labelling and microscopy

*Rrn3*^*wt/wt*^*-*, *Rrn3*^*fl/fl*^*-*, and *UBF*^*fl/fl*^*/ER-Cre*^*+/+*^*/p53*^*-/-*^ MEFs were grown on poly-lysine coated coverslips and treated with 4-HT to induce full inactivation of floxed genes (72h time point), and gene inactivation monitored in parallel by genotyping and measurement of protein levels. 1mM 5-ethynyl uridine (EU, Molecular Probes / Thermo Fisher) was added to the culture medium and cultures incubated for 1h at 37^°^C. Cells were then washed with PBS, fixed in 4% paraformaldehyde (PFA) in PBS for 15 minutes and permeabilized with 0.5% Triton in PBS for 5 minutes. EU incorporation was revealed following the manufacturers protocol (Click-iT RNA HCS, Alexa488). Cells were then incubated with the indicated primary antibodies for 1h in PBS plus 5% BSA or Maxblock (Active Motif) and subsequently with AlexaFluor 568/647 conjugated anti-rabbit or -mouse secondary antibodies (Molecular Probes / Thermo Fisher) and counterstained with DAPI. After mounting in 50% glycerol/50% 0.2 M Na-glycine, 0.3 M NaCl, image stacks were acquired using a Leica SP5 II scanning confocal microscope and formatted with Volocity software (Perkin Elmer Improvision).

### Ethics statement

All animal care and animal experiments were conducted in accordance with the guidelines provided by the Canadian Council for Animal Protection, under the surveillance and authority of the institutional animal protection committees of Laval University and the CHU de Québec. The specific studies described were performed under protocol #2011–054, 2014–100, and 2014–101 examined and accepted by the “Comité de protection des animaux du CHU de Québec”. This ensured that all aspects of the work were carried out following strict guidelines to ensure careful, consistent and ethical handling of mice.

## Supporting information

S1 Fig GC content of the rDNA and UBF binding. The normalized map profile of UBF binding across the full rDNA repeat unit and the percent G+C content of the rDNA calculated over 50bp non-overlapping sliding windows [93]. A scale map of the rDNA sequence elements is given below the panel.
(PDF)

S2 Fig Deletion of the *Rrn3-Tif1a* gene arrests mouse development during early cleavage divisions. A) Genotyping of embryos and live pups from matings of *Rrn3-Tif1a*^*+/-*^ mice either before or after (BL6) backcrossing to the C57BL/6 background. B) Example images of E3.5 embryos obtained from the matings. C) Double heterozygous *Ubf*^*+/-*^*/Rrn3-Tif1a*^*+/-*^ mice are both viable and generated with the expected Mendelian frequency in matings between *Ubf*^*+/-*^ *and Rrn3-Tif1a*^*+/-*^ mice. It was previously found that homozygous deletion of the mouse Fibrillarin (*Fbl*), RPI second largest subunit (*Rpa135*/*Rpo1-2/Polr1b*) or Upstream Binding Factor (*Ubf/Ubtf*) genes all cause developmental arrest during the cleavage divisions and well before the blastula stage [35, 94, 95]. This is consistent with the activation of the rRNA genes at or soon after the 2-cell stage [60, 96]. In contrast, *Rrn3-Tif1a*^*-/-*^ mouse embryos were reported not to arrest development until E9.5 at which point they clearly displayed axis formation, tissue differentiation and the beginnings of organogenesis [45]. By E9.5 zygotic transcription would normally be expected to have increased rRNA levels over 1000 fold [46, 47]. Thus, these data suggested that Rrn3-TIF1A might either not strictly be essential or be partly redundant with some other factor. While establishing *Rrn3-Tif1a* conditional cell lines carrying the *Tif1a*^*flox*^ allele created by Yuan et al., we also generated mice carrying the same *Rrn3-Tif1a-null* allele studied by these authors. When progeny from the *Rrn3-TIF1A*^*+/-*^ mice were analyzed we found that null embryos in fact arrested during the cleavage divisions as un-compacted morulae. The same result was obtained after extensive backcrossing to C57BL/6, the mouse strain used in the original publication. Since our *Rrn3-Tif1a*^*-/+*^ mouse lines were extensively backcrossed to remove any transgenes used in recombining the *Rrn3-Tif1a* ^*flox*^ allele, we presently have no explanation for the discrepancy with the previous study. We concluded that, despite previous data to the contrary, Rrn3-TIF1a, like UBF and RPI, is essential in mouse soon after the normal onset of rRNA gene activity. This strongly argues that the mouse Rrn3 is indeed an essential and non-redundant part of the RPI transcription machinery. It further suggests that the fertilised oocyte does not contain a significant amount of maternal Rrn3 message or protein or, as has been suggested for UBF [97], that these are subject to rapid degradation during the first few cleavage divisions.
(PDF)

S3 Fig Functional analysis of UBF and Rrn3 inactivation in conditional MEFs. A) and D) show maps of the wild type (wt), conditional (flox) and deleted (Δ) Rrn3 and UBF gene alleles. B) and E) show typical time courses (Hours post 4-HT) of Rrn3 and UBF gene deletion determined by PCR genotyping (Genotype), in parallel with corresponding protein levels for each factor. C) The anti-Rrn3 antibody generated in our laboratory and used for ChIP analyses revealed a single endogenous Rrn3 polypeptide that corresponded in mobility with the known Rrn3 species (Genbank XP_156394 and NP_001034610) expressed by transient transfection (Exogenous). F) Time courses of relative 47S rRNA synthesis rates in conditional and wild type Rrn3 and UBF MEFs post 4-HT treatment as determined by metabolic labeling (Materials and Methods). G) Colony forming assay for *Rrn3*^*fl/fl*^*-*, and *UBF*^*fl/fl*^*/ER-Cre*^*+/+*^*/p53*^*-/-*^ (*flox/flox*) and matched *Rrn3*^*+/+*^*-*, and *UBF*^*+/+*^*/ER-Cre*^*+/+*^*/p53*^*-/-*^ (*wt/wt*) MEFs. Cultures were standardly treated with 4-HT, replated 48h later and crystal violet staining of resulting cell colonies determined at 144h post 4-HT treatment. The assay showed that around 4% of *Rrn3*^*fl/fl*^ cells, but less than 1% of *Ubf*^*fl/fl*^ cells, were able to form colonies and hence retained a functional *Rrn3* or *Ubf* after the 4-HT treatment.
(PDF)

S4 Fig TTF1 may bind at low level throughout the 47S gene body during active transcription. The normalized ChIP-Seq profiles for TTF1 before and after (72h post 4-HT) Rrn3 or UBF gene deletion and compared to the same data for RPI. The data are similar to those in Figs 2 and 3, but the vertical scale has been magnified to reveal the low level enrichments.
(PDF)

S5 Fig UBF determines the activated state of the rDNA chromatin. A) The mapped position of the hybridization probes relative to the start of the 47S rRNA are indicated above a diagram of the rDNA repeat. B) The profiles of increasing MNase cleavage of chromatin from UBF conditional MEFs before or after (72h post 4-HT) inactivation of the gene, and C) from Rrn3 wild type and conditional (floxed) MEFs after 72h of treatment with 4-HT. The total DNA cleavage ladders were revealed by ethidium bromide (EtBr) staining, and the cleavage ladders within the IGS and the 47S gene body were revealed by hybridization with the corresponding probes shown in A). The positions of mono- (1), di- (2), etc nucleosomes are indicated.
(PDF)

S6 Fig H3K9ac, H3K36me3 and H3K27ac histone modifications also map to the Enhancer Boundary Complex. The RPM profiles for H3K9ac, H3K36me3 and H3K27ac realigned from public data (ENCODE GSE32218) over the Promoter and Enhancer region of the mouse rDNA are shown in comparison with the CTCF and H3K4me3 enrichment profiles established in the present study.
(PDF)

S7 Fig Comparative ChIP-QPCR analyses show Enhancer Boundary Complex components are retained after Rrn3 and UBF deletion. A) Positions of QPCR amplicons relative to rDNA sequence motifs. B) and C) ChIP-QPCR analyses at 3 days post 4HT treatment respectively for *Rrn3*^*fl/fl*^*-* and *Rrn3*^*+/+*^*/ER-Cre*^*+/+*^*/p53*^*-/-*^ and *Ubf*^*fl/fl*^*-* and *Ubf*^*+/+*^*/ER-Cre*^*+/+*^*/p53*^*-/-*^ MEFs. The data show the results for Histone H3, H3-K4me3, CTCF and either Rrn3 or UBF, the factor targeted by deletion.
(PDF)
