# Supplementary figures and images for: A unique enhancer boundary complex on the mouse ribosomal RNA genes persists after loss of Rrn3 or UBF and the inactivation of RNA polymerase I transcription
Source: PLoS Genet. 2017 Jul 17;13(7):e1006899. doi: 10.1371/journal.pgen.1006899 (PMC5536353; doi:10.1371/journal.pgen.1006899)

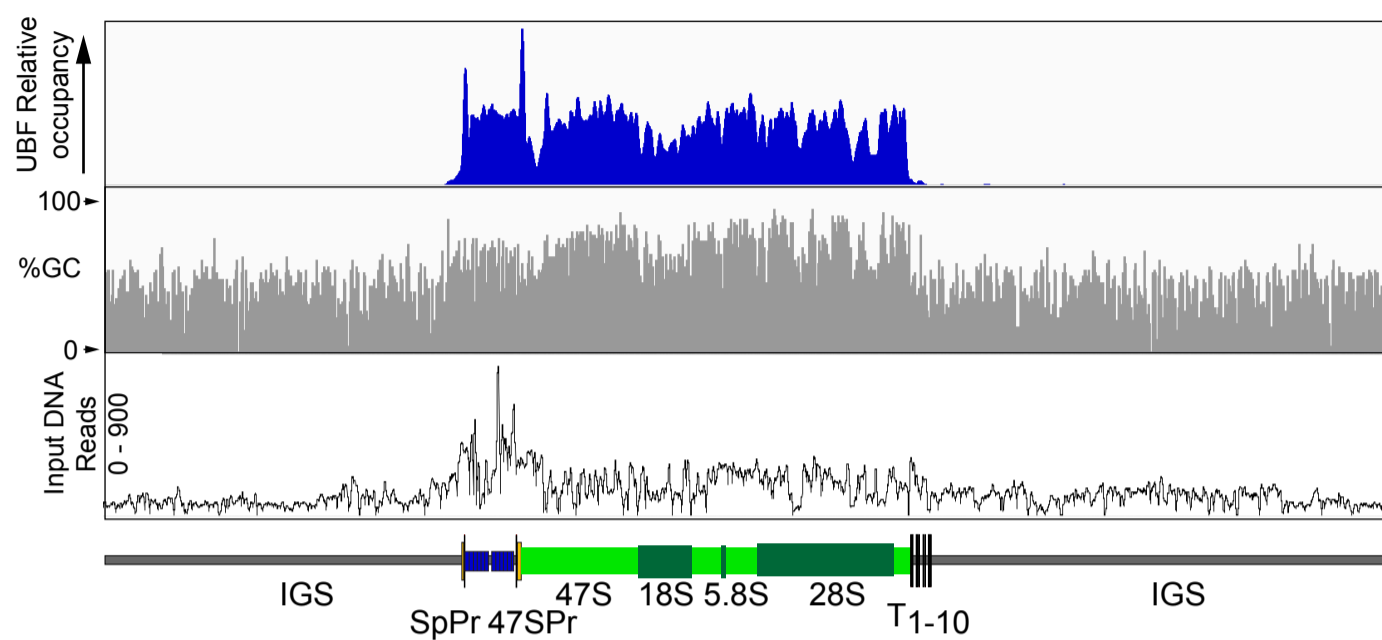

Supplement: S1 Fig — The normalized map profile of UBF binding across the full rDNA repeat unit and the percent G+C content of the rDNA calculated over 50bp non-overlapping sliding windows [93]. A scale map of the rDNA sequence elements is given below the panel. (PDF) [file pgen.1006899.s001.pdf]

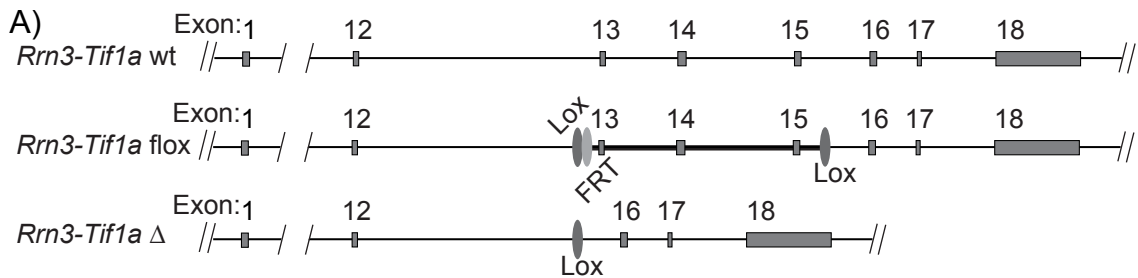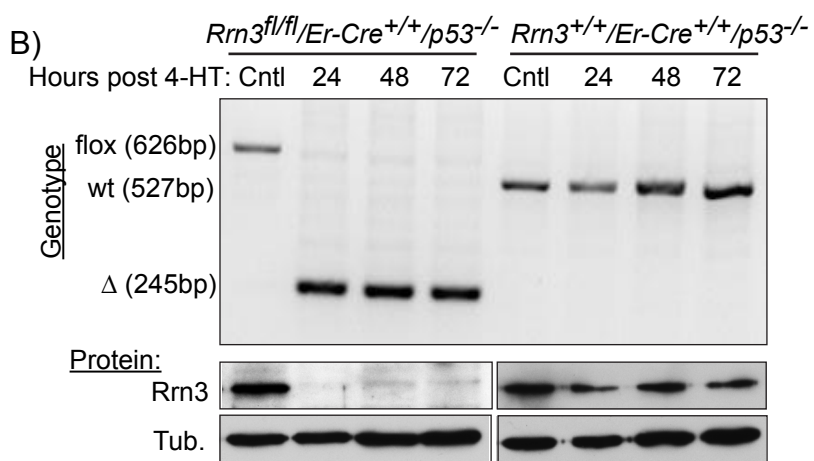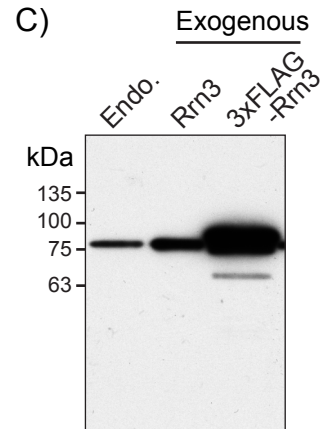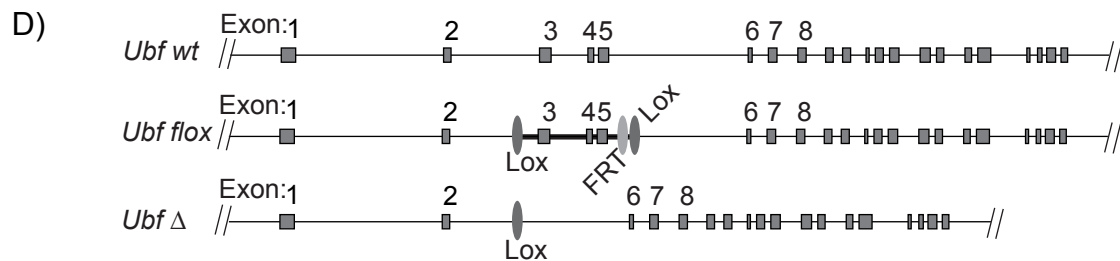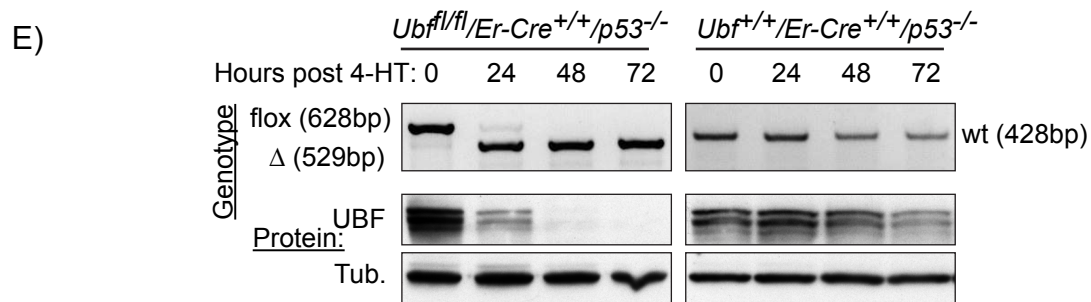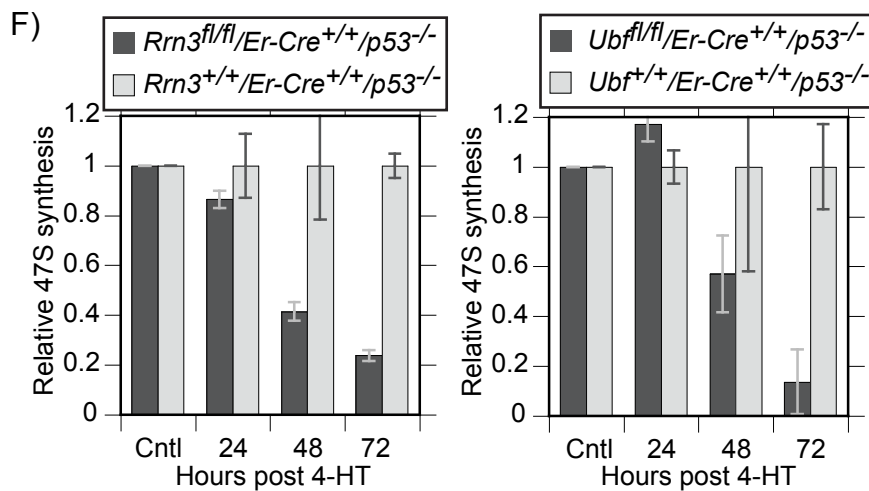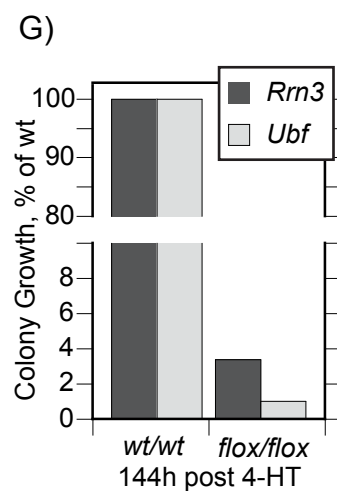

Supplement: S3 Fig — A) and D) show maps of the wild type (wt), conditional (flox) and deleted (Δ) Rrn3 and UBF gene alleles. B) and E) show typical time courses (Hours post 4-HT) of Rrn3 and UBF gene deletion determined by PCR genotyping (Genotype), in parallel with corresponding protein levels for each factor. C) The anti-Rrn3 antibody generated in our laboratory and used for ChIP analyses revealed a single endogenous Rrn3 polypeptide that corresponded in mobility with the known Rrn3 species (Genbank XP_156394 and NP_001034610) expressed by transient transfection (Exogenous). F) Time courses of relative 47S rRNA synthesis rates in conditional and wild type Rrn3 and UBF MEFs post 4-HT treatment as determined by metabolic labeling (Materials and Methods). G) Colony forming assay for Rrn3fl/fl-, and UBFfl/fl/ER-Cre+/+/p53-/- (flox/flox) and matched Rrn3+/+-, and UBF+/+/ER-Cre+/+/p53-/- (wt/wt) MEFs. Cultures were standardly treated with 4-HT, replated 48h later and crystal violet staining of resulting cell colonies determined at 144h post 4-HT treatment. The assay showed that around 4% of Rrn3fl/fl cells, but less than 1% of Ubffl/fl cells, were able to form colonies and hence retained a functional Rrn3 or Ubf after the 4-HT treatment. (PDF) [file pgen.1006899.s003.pdf]

Genotype:-

IP:-

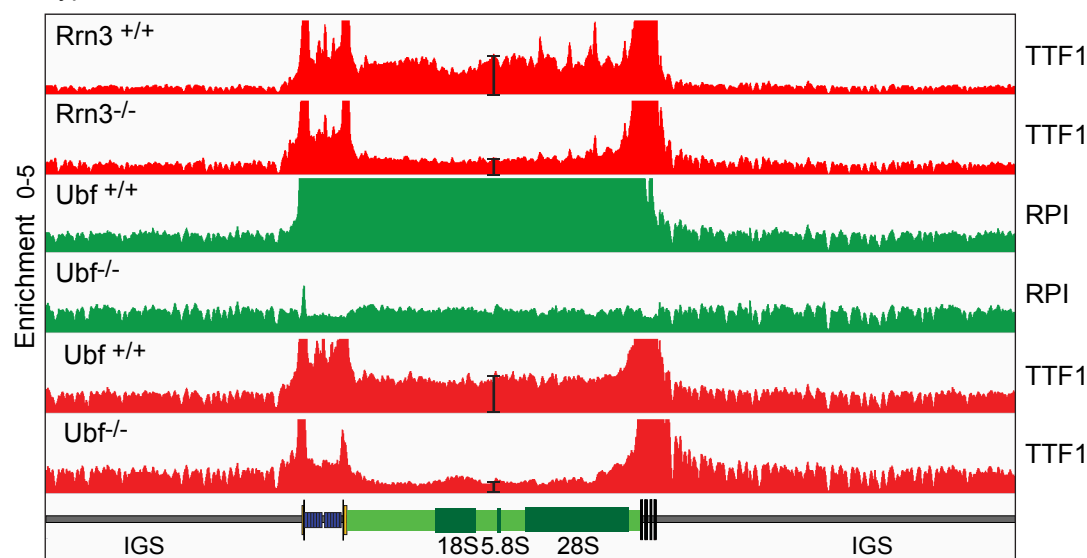

Supplement: S4 Fig — The normalized ChIP-Seq profiles for TTF1 before and after (72h post 4-HT) Rrn3 or UBF gene deletion and compared to the same data for RPI. The data are similar to those in Figs 2 and 3, but the vertical scale has been magnified to reveal the low level enrichments. (PDF) [file pgen.1006899.s004.pdf]

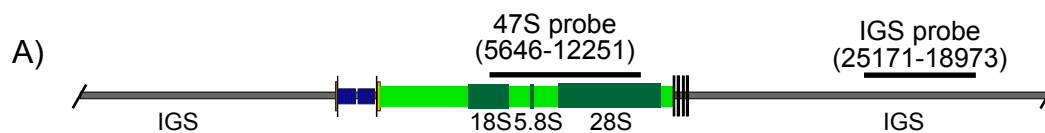

B) UBF inactivation

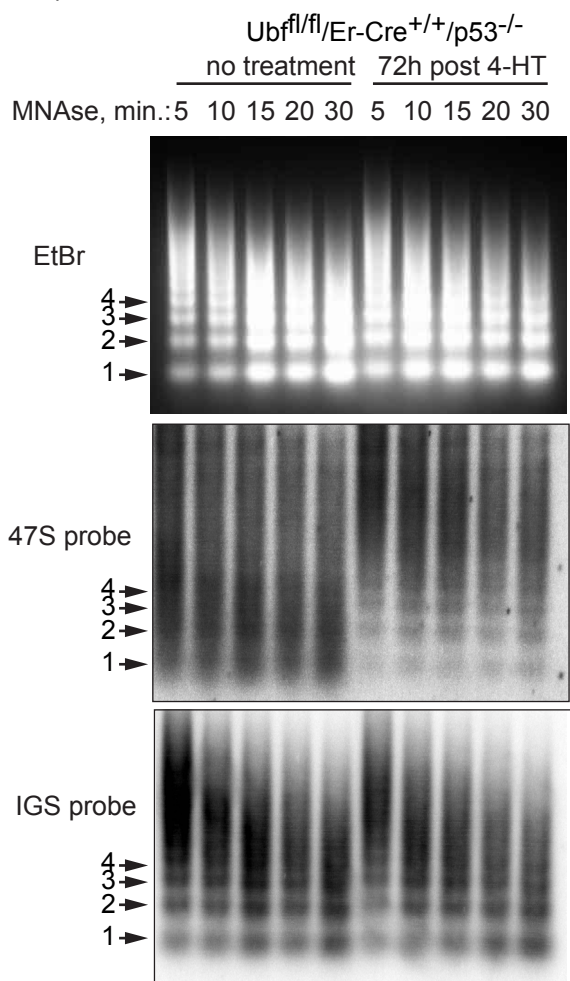

C) Rrn3 inactivation

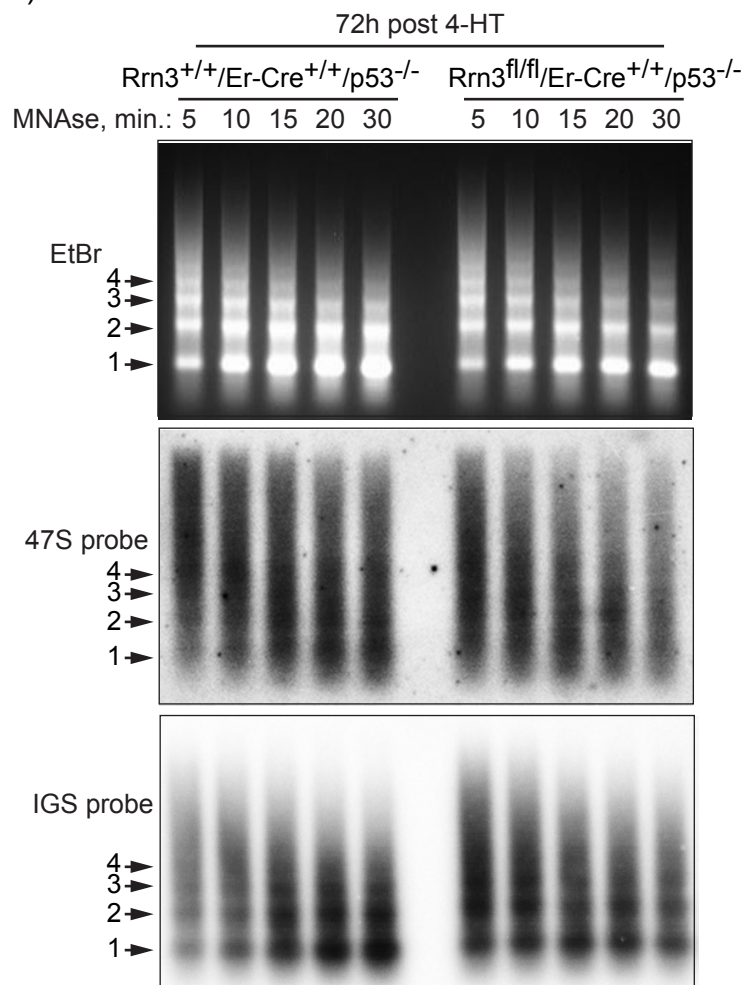

Supplement: S5 Fig — A) The mapped position of the hybridization probes relative to the start of the 47S rRNA are indicated above a diagram of the rDNA repeat. B) The profiles of increasing MNase cleavage of chromatin from UBF conditional MEFs before or after (72h post 4-HT) inactivation of the gene, and C) from Rrn3 wild type and conditional (floxed) MEFs after 72h of treatment with 4-HT. The total DNA cleavage ladders were revealed by ethidium bromide (EtBr) staining, and the cleavage ladders within the IGS and the 47S gene body were revealed by hybridization with the corresponding probes shown in A). The positions of mono- (1), di- (2), etc nucleosomes are indicated. (PDF) [file pgen.1006899.s005.pdf]

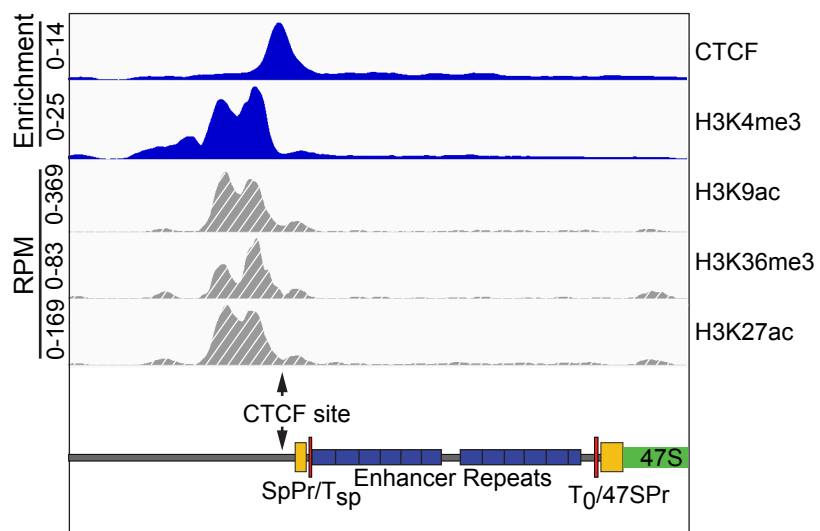

Supplement: S6 Fig — The RPM profiles for H3K9ac, H3K36me3 and H3K27ac realigned from public data (ENCODE GSE32218) over the Promoter and Enhancer region of the mouse rDNA are shown in comparison with the CTCF and H3K4me3 enrichment profiles established in the present study. (PDF) [file pgen.1006899.s006.pdf]

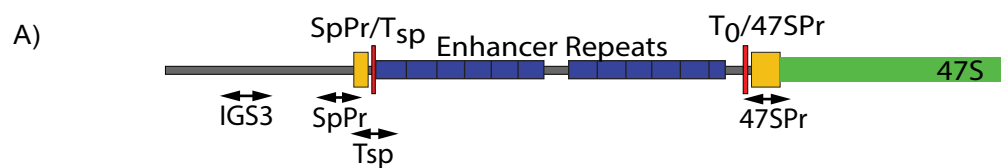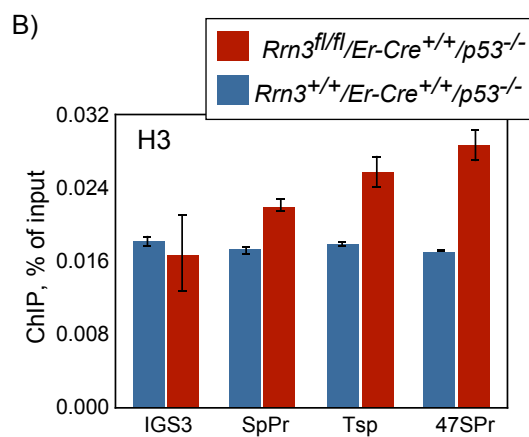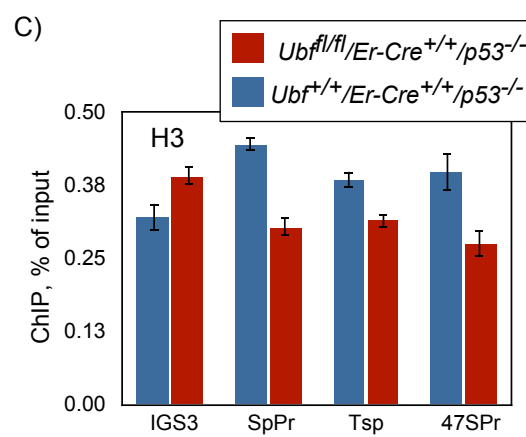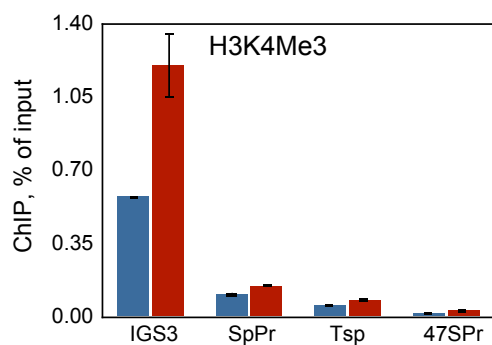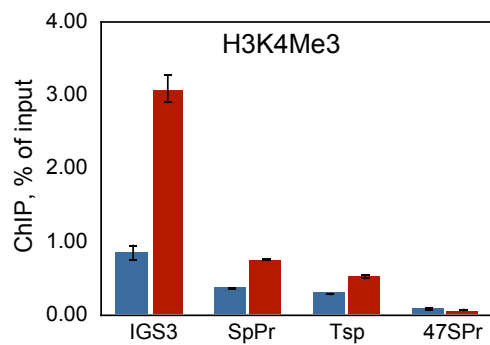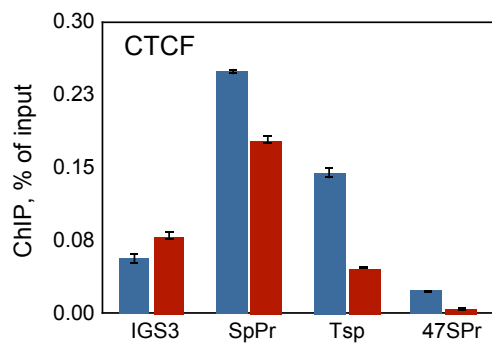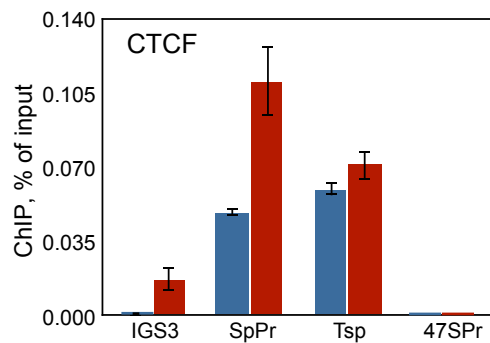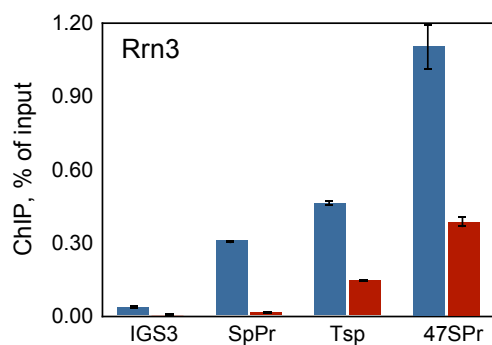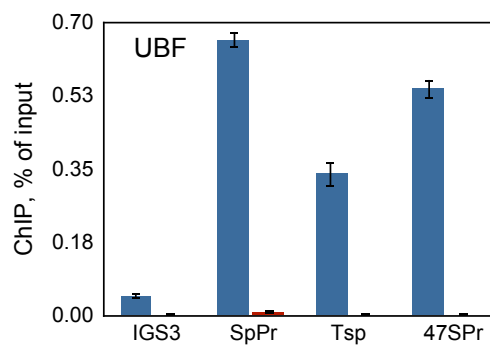

Supplement: S7 Fig — A) Positions of QPCR amplicons relative to rDNA sequence motifs. B) and C) ChIP-QPCR analyses at 3 days post 4HT treatment respectively for Rrn3fl/fl- and Rrn3+/+/ER-Cre+/+/p53-/- and Ubffl/fl- and Ubf+/+/ER-Cre+/+/p53-/- MEFs. The data show the results for Histone H3, H3-K4me3, CTCF and either Rrn3 or UBF, the factor targeted by deletion. (PDF) [file pgen.1006899.s007.pdf]
