# Supplementary material for: A unique enhancer boundary complex on the mouse ribosomal RNA genes persists after loss of Rrn3 or UBF and the inactivation of RNA polymerase I transcription
Source: PLoS Genet. 2017 Jul 17;13(7):e1006899. doi: 10.1371/journal.pgen.1006899 (PMC5536353; doi:10.1371/journal.pgen.1006899)
Supplement: S2 Fig — A) Genotyping of embryos and live pups from matings of Rrn3-Tif1a+/- mice either before or after (BL6) backcrossing to the C57BL/6 background. B) Example images of E3.5 embryos obtained from the matings. C) Double heterozygous Ubf+/-/Rrn3-Tif1a+/- mice are both viable and generated with the expected Mendelian frequency in matings between Ubf+/- and Rrn3-Tif1a+/- mice. It was previously found that homozygous deletion of the mouse Fibrillarin (Fbl), RPI second largest subunit (Rpa135/Rpo1-2/Polr1b) or Upstream Binding Factor (Ubf/Ubtf) genes all cause developmental arrest during the cleavage divisions and well before the blastula stage [35, 94, 95]. This is consistent with the activation of the rRNA genes at or soon after the 2-cell stage [60, 96]. In contrast, Rrn3-Tif1a-/- mouse embryos were reported not to arrest development until E9.5 at which point they clearly displayed axis formation, tissue differentiation and the beginnings of organogenesis [45]. By E9.5 zygotic transcription would normally be expected to have increased rRNA levels over 1000 fold [46, 47]. Thus, these data suggested that Rrn3-TIF1A might either not strictly be essential or be partly redundant with some other factor. While establishing Rrn3-Tif1a conditional cell lines carrying the Tif1aflox allele created by Yuan et al., we also generated mice carrying the same Rrn3-Tif1a-null allele studied by these authors. When progeny from the Rrn3-TIF1A+/- mice were analyzed we found that null embryos in fact arrested during the cleavage divisions as un-compacted morulae. The same result was obtained after extensive backcrossing to C57BL/6, the mouse strain used in the original publication. Since our Rrn3-Tif1a-/+ mouse lines were extensively backcrossed to remove any transgenes used in recombining the Rrn3-Tif1a flox allele, we presently have no explanation for the discrepancy with the previous study. We concluded that, despite previous data to the contrary, Rrn3-TIF1a, like UBF and RPI, is essential in [file pgen.1006899.s002.pdf]

A)

Age (dpc)	Number	*Rrn3(Tif1a)*$^{+/+}$	*Rrn3(Tif1a)*$^{+/-}$	*Rrn3(Tif1a)*$^{-/-}$
3.5	25	6 (24.0%)	14 (56.0%)	5 (20.0%)
3.5 (BL/6)	10	3 (30.0%)	4 (40.0%)	3 (30.0%)
6.5	17	9 (53.0%)	8 (47.0%)	0 (0.0%)
8.5	14	3 (21.4%)	11 (78.6%)	0 (0.0%)
8.5 (BL/6)	9	3 (33.3%)	6 (66.7%)	0 (0.0%)
9.5	36	10 (27.8%)	26 (72.2%)	0 (0.0%)
9.5 (BL/6)	29	6 (20.7%)	23 (79.3%)	0 (0.0%)
10.5	8	3 (37.5%)	5 (62.5%)	0 (0.0%)
Pups	70	22 (31.4%)	48 (68.6%)	0 (0.0%)
Pups (BL/6)	21	5 (23.8%)	16 (76.2%)	0 (0.0%)

B)

Rrn3(Tif1a)$^{+/+}$ *Rrn3(Tif1a)*$^{+/-}$ *Rrn3(Tif1a)*$^{-/-}$

E3.5

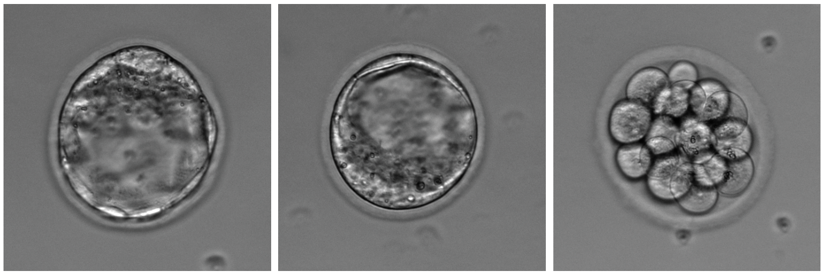

C)

Ubf$^{+/-}$*Rrn3*$^{+/-}$

Pups	Genotype								
	+/+ +/+	+/+ +/-	+/+ -/-	+/- +/+	+/- +/-	+/- -/-	-/- +/+	-/- +/-	-/- -/-
Number:	1 (3.6%)	8 (28.6%)	9 (0%)	10 (21.4%)	13 (46.4%)	0 (0%)	0 (0%)	0 (0%)	0 (0%)
Expected:	6.25%	12.50%	6.25%	12.50%	25.00%	12.50%	6.25%	12.50%	6.25%
